# Effective Radiosensitization of HNSCC Cell Lines by DNA-PKcs Inhibitor AZD7648 and PARP Inhibitors Talazoparib and Niraparib

**DOI:** 10.3390/ijms25115629

**Published:** 2024-05-22

**Authors:** Jacob Mentzel, Laura S. Hildebrand, Lukas Kuhlmann, Rainer Fietkau, Luitpold V. Distel

**Affiliations:** 1 Department of Radiation Oncology, Universitätsklinikum Erlangen, Friedrich-Alexander-Universität Erlangen-Nürnberg (FAU), 91054 Erlangen, Germany; jacobmentzel@gmx.de (J.M.); laura.hildebrand@uk-erlangen.de (L.S.H.); lukas.kuhlmann@uk-erlangen.de (L.K.); rainer.fietkau@uk-erlangen.de (R.F.); 2 Comprehensive Cancer Center Erlangen-Europäische Metropolregion Nürnberg (CCC ER-EMN), 91054 Erlangen, Germany

**Keywords:** HNSCC, DNA damage response inhibitor, PARP inhibitor, DNA-PK inhibitor, kinase inhibitor, radiosensitivity, ionizing radiation, cell lines

## Abstract

(1) Head and neck squamous cell carcinoma (HNSCC) is common, while treatment is difficult, and mortality is high. Kinase inhibitors are promising to enhance the effects of radiotherapy. We compared the effects of the PARP inhibitors talazoparib and niraparib and that of the DNA-PKcs inhibitor AZD7648, combined with ionizing radiation. (2) Seven HNSCC cell lines, including Cal33, CLS-354, Detroit 562, HSC4, RPMI2650 (HPV-negative), UD-SCC-2 and UM-SCC-47 (HPV-positive), and two healthy fibroblast cell lines, SBLF8 and SBLF9, were studied. Flow cytometry was used to analyze apoptosis and necrosis induction (AnnexinV/7AAD) and cell cycle distribution (Hoechst). Cell inactivation was studied by the colony-forming assay. (3) AZD7648 had the strongest effects, radiosensitizing all HNSCC cell lines, almost always in a supra-additive manner. Talazoparib and niraparib were effective in both HPV-positive cell lines but only consistently in one and two HPV-negative cell lines, respectively. Healthy fibroblasts were not affected by any combined treatment in apoptosis and necrosis induction or G2/M-phase arrest. AZD7648 alone was not toxic to healthy fibroblasts, while the combination with ionizing radiation reduced clonogenicity. (4) In conclusion, talazoparib, niraparib and, most potently, AZD7648 could improve radiation therapy in HNSCC. Healthy fibroblasts tolerated AZD7648 alone extremely well, but irradiation-induced effects might occur. Our results justify in vivo studies.

## 1. Introduction

Head and neck squamous cell carcinoma (HNSCC) is the eighth most common cancer worldwide, with over 878,000 new cases and over 444,000 deaths in 2020 [1]. Its 5-year survival rate is approximately 50%, ranging from 80% for stage I to as low as 20% for stages III/IV [2]. The association of late diagnosis with low survival is especially problematic, since no screening strategy has proved to be effective to this day [3]. The major risk factors are consumption of tobacco and alcohol, environmental pollutants, the chewing of areca nut products and infection with the Epstein-Barr virus (EBV) and the human papilloma virus (HPV) [3,4,5]. While the use of tobacco and alcohol is mainly associated with cancers of the oral cavity and the larynx and the use of areca nut products is also linked to oral cavity cancer, infections with HPV and EBV are associated with oropharyngeal and nasopharyngeal cancer, respectively [3]. Tobacco and alcohol consumption not only increase the HNSCC risk on their own but also, multiplicatively, in heavy consumers of both, by 38-fold [6]. The chewing of areca nut products makes for high HNSCC incidence in some Asian countries such as India, China and Papua New Guinea [1,7]. Viral infections with EBV and, most prominently, with HPV play an important role in HNSCC development, being responsible for 70% of new cases in Europe and North America [4,8,9]. HNSCC can be divided into HPV-associated (HPV-positive) and non-HPV-associated (HPV-negative) disease. As the incidence of HPV-positive HNSCC has risen [4], there is growing hope that, as in cervical cancer [10], primary prevention with HPV vaccination programs might drastically decrease HPV-positive HNSCC incidence in the future. This could potentially shift the focus to HPV-negative HNSCC, which is generally more resistant to the current treatment modalities, including being more radioresistant and thus associated with worse prognosis, even in lower TNM (tumor, node, metastasis) stages [9,11,12]. Nevertheless, radiation therapy plays an important role in the treatment of HNSCC [3,13].

Therefore, finding ways to make HNSCC more radiosensitive is of great interest. Irradiation induces DNA damage, including single-strand breaks (SSBs) and double-strand breaks (DSBs) [14]. DSBs are considered the most lethal DNA lesion, leading to cell cycle arrest or cell death if left unrepaired [14,15,16]. The DNA damage response (DDR) system is responsible for repairing DNA damage. A method to enhance radiosensitivity is to inhibit DDR and simultaneously induce DNA damage, for example, through irradiation [17,18]. The DNA-damage which then cannot be repaired accumulates, ultimately leading to cell death [19,20]. Most SSBs are repaired, the reparation process being carried out by the SSB repair system [14,21], while repairing DSBs is more complex. The two major systems of DDR to repair DSBs are the highly precise homologous recombination (HR) and non-homologous end-joining (NHEJ), which is more error-prone [17,22,23], since it ligates broken DNA ends together without a template [15,16,17,22,24,25]. Tumor cells are often HR-deficient and heavily rely on NHEJ for DSB repair, since impaired DDR and especially DSB repair results in genomic instability, which is a hallmark of cancer [19,21,26,27,28,29]. Therefore, combining irradiation with the inhibition of either the SSB repair system or NHEJ is a promising method for treating cancer, including HNSCC. In contrast to cancer cells, healthy cells are normally HR-sufficient and can tolerate a certain amount of irradiation-induced DNA damage [29]. In theory, this could limit unwanted side effects.

The protein poly(ADP-ribose) polymerase (PARP) is a sensor for DNA damage. Despite PARP1 being first isolated in 1971, PARP’s involvement in DSB repair, especially alternative-NHEJ, and other cellular processes beyond DNA damage repair, is still the subject of active debate [21,30,31]. Meanwhile, its role in SSB repair is much better understood. PARP1 detects SSBs, then binds to the DNA, resulting in a conformational change that activates PARP1. Activated PARP1 cleaves off ADP-ribose from NAD+ multiple times, creating poly(ADP-ribose) chains (PAR chains) and attaching them to nuclear proteins. The chains are negatively charged, thus electrostatically attracting XRCC1 and repulsing PARylated histones and PARP1 [21,31,32]. The SSB repair is then continued without PARP1. PARP1 might also be involved in the repair of damaged bases, in a process called base excision repair (BER), although existing data are contradictory [32,33,34,35,36,37]. In the process of base excision, an SSB is created [14]. The repair pathway of these self-created SSBs is closely related to regular SSB repair [32].

PARP inhibition impairs SSB repair, although not all mechanisms involved are fully understood [38]. Unrepaired SSBs can turn into DSBs in the S-phase, which in turn occurs more often in rapidly proliferating cells [14,17]. Apart from simply blocking PARP´s ability to repair SSBs, PARP inhibition also prevents PARP from dissociating from the DNA in a process called PARP-trapping, which leads to the collapse of the replication fork and subsequently results in replication-dependent DSBs [31,38,39]. In HR-deficient cells, especially in the BRCA-deficient breast cancer cells, there is a dramatically increased sensitivity to PARP inhibitors [40]. In these cells, the error-prone NHEJ is responsible for repairing DSBs, resulting in increased rates of unrepaired DSBs and cell death [31,41]. Multiple PARP inhibitors (PARPis; singular: PARPi) have already been market-approved in different gynecological tumor entities [42,43,44,45,46,47], and preclinical evidence indicates the radiosensitizing effects of PARPis in HNSCC as well [48,49,50,51,52].

The DNA-dependent protein kinase (DNA-PK) catalytic subunit (DNA-PKcs) is essential for NHEJ [16,21,53]. DNA-PK binds to previously detected DNA DSBs and is activated by autophosphorylation. This results in conformational and positional changes of DNA-PK, enabling end-processing and ligation enzymes to repair the DSB [14,21]. NHEJ occurs in all cell cycle phases [14,54]. DNA-PKcs, which is a member of the phosphatidylinositol 3-kinase-related kinase (PIKK) family, also has some lesser known functions critical to cellular survival and proliferation, such as regulating transcription, progressing through cell cycle or maintaining telomers [55,56,57].

The PIKK family also includes Ataxia Teleangiectasia Mutated (ATM) and Ataxia Teleangiectasia Related (ATR), which are similar essential proteins involved in the DNA damage response. A further protein is mTOR, as a regulator of cell cycle control and proliferation. Because of their central role in the DNA damage response, they are interesting targets for kinase inhibitors [58].

Both kinase inhibitor types share the characteristic that they have no effect on cells without DNA damage. Since cancer cells tend to have higher levels of DNA damage due to oxidative stress, these cells are more likely to be targeted by the inhibitors [59]. However, with IR, DNA damage can be specifically induced in cancers at much higher levels, making the inhibitors more effective.

In the context of radiosensitization, the inhibition of NHEJ through DNA-PKcs inhibition is especially relevant, since HR-deficient cells are dependent on NHEJ to repair DSBs and, in turn, to survive [19,21]. Combining a DNA-PKcs inhibitor (DNA-PKcsi) with ionizing radiation (IR) is therefore promising in enhancing radiosensitivity in tumors [15,60]. Some DNA-PKcsis have previously shown radiosensitizing capabilities in HNSCC cells both in vitro and in vivo [61,62,63,64,65], and several clinical trials are underway, involving DNA-PKcsis plus IR in solid tumors, in some cases HNSCC (NCT03907969, NCT02516813, NCT04533750), and also evaluating safety and efficacy in solid tumors (NCT01353625) [66].

In this study, our goal was to evaluate whether the PARPis talazoparib and niraparib, as well as the highly selective DNA-PKcsi AZD7648, radiosensitize HNSCC cell lines in vitro and, therefore, may have the potential to improve radiation therapy in HNSCC in the future. Seven HNSCC cell lines were investigated, two of which were HPV-positive, while five were HPV-negative, which are generally more radioresistant [9,11,12]. We also included two healthy fibroblast cell lines to estimate the severity of the side effects that can be expected. This is useful, since radiation therapy can never completely spare healthy tissue in a clinical setting, and by investigating treatment effects on healthy fibroblasts representative of healthy tissue exposed to the treatment, the risk of potential side effects can be estimated. We assessed apoptotic and necrotic cell death induction and cell cycle arrest in the G2/M-phase using flow cytometry. Additionally, we performed colony formation assays as the gold-standard for evaluating in vitro radiosensitivity [67,68]. The endpoints that were particularly of interest were cell death, the G2/M-phase arrest and cell inactivation, since, combined, they provide a comprehensive image of the effects and potential of a treatment. Interesting from a clinical perspective was primarily whether combining a kinase inhibitor (KI) with IR is more effective than IR alone. Supra-additivity was also calculated.

## 2. Results

**Figure 1:**
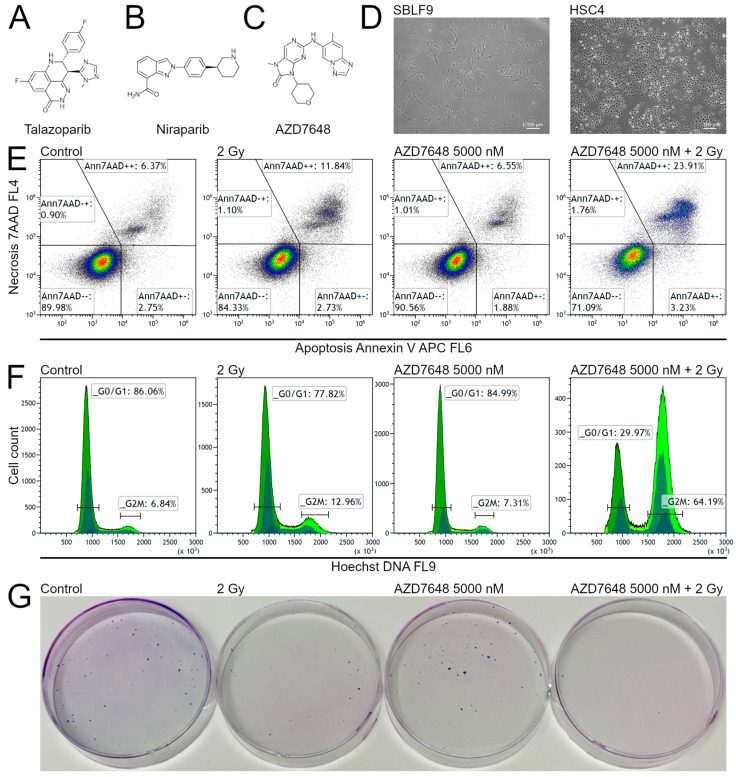
Kinase Inhibitors, cell lines and methods. (**A**) Talazoparib (50 nM), (**B**) niraparib (2500 nM) and (**C**) AZD7648 (5000 nM) were evaluated, each both with and without IR. (**D**) Microscopic images of SBLF9 and HSC4 are depicted representatively for the two healthy fibroblast cell lines and the seven HNSCC cell lines examined. The white bar equals 100 µm. (**E**) Representative scatter plot of the flow cytometric measurement of apoptosis and necrosis induction by Annexin V and 7AAD in UM-SCC-47. (**F**) Representative histograms of the Hoechst 33342-stained and flow cytometrically analyzed cell cycle distribution in UM-SCC-47 cell line. (**G**) Representative Petri dishes for colony formation assay containing Cal33 colonies. For all methods, the displayed samples are control, 2 Gy, AZD7648 5000 nM and AZD7648 5000 nM + 2 Gy. For all three methods, half of the samples were irradiated with 2 Gy IR, 3 h after the inhibitors were added to all samples. After a further 48 h, the inhibitors were removed, and either flow cytometric measurements were performed or standard medium was added to the colony formation assay.

Three KIs that interfere with DNA repair were evaluated for efficacy in combination with IR. Two of the compounds were the PARPis talazoparib (Figure 1A) and niraparib (Figure 1B). The third was the DNA-PKcsi AZD7648 (Figure 1C). Nine cell lines, including two fibroblast cell lines as normal tissue cell lines and seven tumor cell lines from HNSCC, were used (Figure 1D).

Cell death and G2/M-phase arrest were analyzed by flow cytometry using Annexin V to estimate apoptotic death, 7AAD to estimate necrotic death (Figure 1E) and Hoechst to estimate cell cycle distribution (Figure 1F). Colony formation was performed in Petri dishes and stained with methylene blue (Figure 1G). To characterize potential synergistic effects of the combined treatments, we calculated supra-additive effects, which indicate that the effect of a combined treatment exceeds the additive effects of the inhibitor treatment and the IR treatment. We used the two-tailed Mann–Whitney U test, as described in more detail in the Materials and Methods Section, to ensure that the supra-additive effects were statistically significant.

### 2.1. Induction of Apoptotic- and Necrotic Death Varies Widely between Different Cell Lines

**Figure 2:**
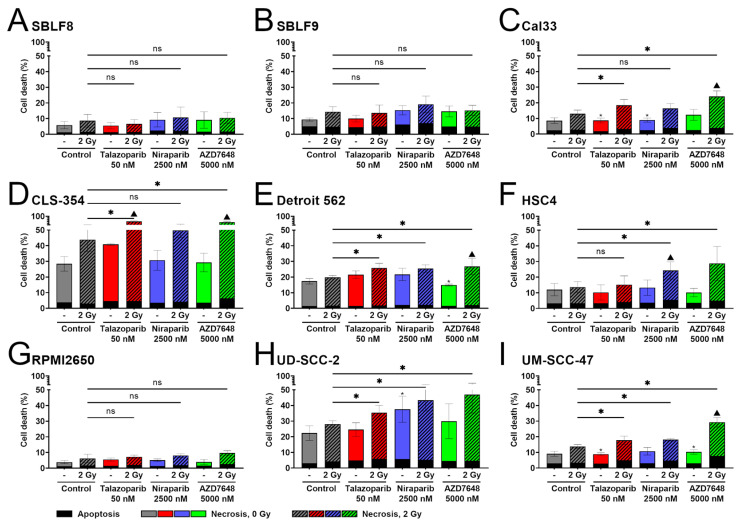
Flow cytometry analysis of apoptosis and necrosis. Healthy fibroblast cells (**A**) SBLF8, (**B**) SBLF9, HPV-negative HNSCC cells, (**C**) Cal33, (**D**) CLS-354, (**E**) Detroit 562, (**F**) HSC4, (**G**) RPMI2650 and HPV-positive HNSCC cells, (**H**) UD-SCC-2, (**I**) UM-SCC-47 were treated either with talazoparib (50 nM), niraparib (2500 nM) or AZD7648 (5000 nM) or combined with 2 Gy IR. After 48 h, apoptosis (Annexin V) and necrosis (7AAD) were measured using flow cytometry. Apoptosis is presented in black, necrosis is color-coded for different inhibitors, and irradiated samples are hatched. Each value represents mean ± SD (*n* ≥ 4). Combined treatments and KI alone were compared to IR alone, and significant changes from IR alone are indicated for combined treatments by asterisks above the corresponding line and for KI alone by small asterisks above the respective bar. The abbreviation “ns” above the corresponding line represents changes that are not significant. A triangle represents supra-additivity. Significance was determined using a two-tailed Mann–Whitney U test with * *p* ≤ 0.05.

In the following analysis, apoptosis and necrosis rates are summarized as cell death. If not specified differently, the treatments are compared to the mono-treatment with IR. In the two healthy fibroblast cell lines, SBLF8 and SBLF9, none of the three KIs, nor any treatment with KI plus IR caused a significant difference in cell death compared to the IR mono-treatment (Figure 2A,B). Niraparib and AZD7648 alone slightly increased cell death in SBLF9 compared to the control. The HNSCC cell lines responded heterogeneously to the combined treatments of KI plus IR. In the two HPV-positive HNSCC cell lines, UD-SCC-2 and UM-SCC-47, as well as in the HPV-negative Detroit 562, the three combined treatments, talazoparib plus IR, niraparib plus IR and AZD7648 plus IR, all resulted in a significant increase in cell death compared to the IR mono-treatment (Figure 2E,H,I). Notably, the niraparib mono-treatment also increased the cell death rate in UD-SCC-2. In RPMI2650, one of this study’s five HPV-negative HNSCC cell lines, there was no significant difference between any treatment and the IR mono-treatment (Figure 2G). The remaining three HPV-negative HNSCC cell lines, Cal33, CLS-354 and HSC4, responded inconsistently to the three combined treatments (Figure 2C,D,F). Talazoparib plus IR resulted in a clear increase in cell death compared to the IR mono-treatment in Cal33 and CLS-354, but not in HSC4. AZD7648 plus IR increased cell death in all three cell lines, and niraparib plus IR did so only in HSC4, but not in Cal33 and CLS-354. It is worth mentioning that significant supra-additive effects were observed for talazoparib plus IR in CLS-354, for niraparib plus IR in HSC4, as well as for AZD7648 plus IR in four out of seven HNSCC cell lines, namely Cal33, CLS-354, Detroit 562 and UM-SCC-47. Further data on supra-additive effects on the induction of apoptosis and necrosis are provided in Supplementary Table S1.

In addition to cell death, cell cycle distribution and the proportion of cells in the G2/M-phase are important, as the cells there are particularly sensitive to radiation [69,70]. Since a pronounced G2/M block has a favorable effect on daily fractionated irradiation, the proportion of cells in G2/M was examined next.

### 2.2. Combined Treatment Causes a Pronounced G2/M Arrest in the Tumor Cell Lines

**Figure 3:**
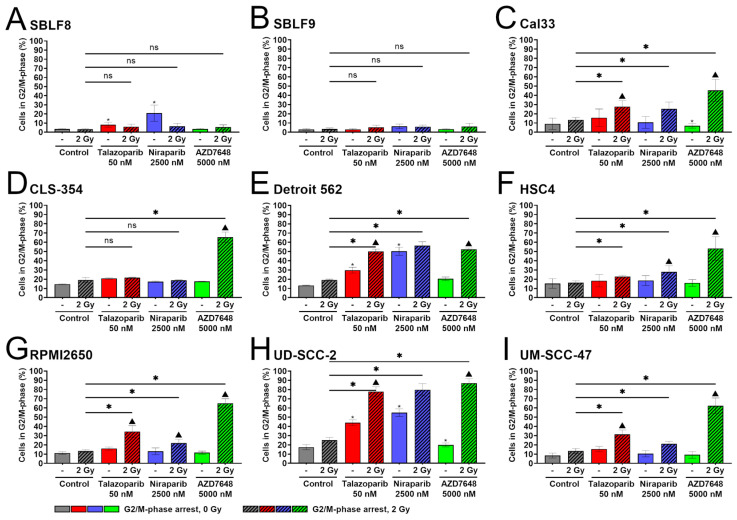
Flow cytometry analysis of cell cycle distribution. Fraction of G2/M-phase arrest. Healthy fibroblast cells (**A**) SBLF8, (**B**) SBLF9, HPV-negative HNSCC cells, (**C**) Cal33, (**D**) CLS-354, (**E**) Detroit 562, (**F**) HSC4, (**G**) RPMI2650 and HPV-positive HNSCC cells, (**H**) UD-SCC-2, (**I**) UM-SCC-47 were treated either with talazoparib (50 nM), niraparib (2500 nM) or AZD7648 (5000 nM) or combined with 2 Gy IR. Incubation lasted for 48 h, and afterward, cells were stained with Hoechst, and cell cycle was analyzed using flow cytometry. Different inhibitors are displayed in color, and irradiated samples are hatched. Each value represents mean ± SD (*n* ≥ 4). Combined treatments and KI alone were compared to IR alone, and significant changes from IR alone are indicated for combined treatments by asterisks above the corresponding line and for KI alone by small asterisks above the respective bar. The abbreviation “ns” above the corresponding line represents changes that are not significant. A triangle represents supra-additivity. Significance was determined using a two-tailed Mann–Whitney U test with * *p* ≤ 0.05.

In the two healthy fibroblast cell lines SBLF8 and SBLF9, all three combined treatments of KI plus IR did not result in a distinct increase in G2/M arrest compared to IR mono-treatment (Figure 3A,B). However, mono-treatments with talazoparib, and especially niraparib, both increased G2/M arrest in SBLF8 compared to the control and the IR mono-treatment. Six out of the seven HNSCC cell lines responded with a clear increase in G2/M arrest to all three combined treatments, although the increase was more distinct in some cell lines than others (Figure 3C,E–I). The only exception was CLS-354, in which only AZD7648 plus IR was effective, with a clear G2/M arrest (Figure 3D). The two PARPis, talazoparib plus IR and niraparib plus IR, had no effect on G2/M arrest in CLS-354.

This can be further described by studying whether these observed effects are statistically significantly supra-additive. That was the case in five of six cell lines for talazoparib plus IR, sparing HSC4. For niraparib plus IR, this effect occurred only in the two cell lines HSC4 and RPMI2650. AZD7648 plus IR showed supra-additive effects in all the examined HNSCC cell lines. Further data on supra-additive effects on the induction of G2/M-phase arrest is provided in Supplementary Table S2.

Additionally, mono-treatment with talazoparib and niraparib increased G2/M arrest compared to mono-treatment with IR in SBLF8, Detroit 562 and UD-SCC-2. Although this effect was not as strong as that of the combined treatments in HNSCC cell lines, it was still relevant. In SBLF8, G2/M arrest was higher with talazoparib, and especially with niraparib mono-treatment, than with the respective combined treatments, which did not show a significant increase compared to the IR mono-treatment. Next, the colony-forming assay was performed, as this best reflects the effect of the treatment.

### 2.3. In Colony Formation Assay AZD7648 Plus IR Is the Most Effective

**Figure 4:**
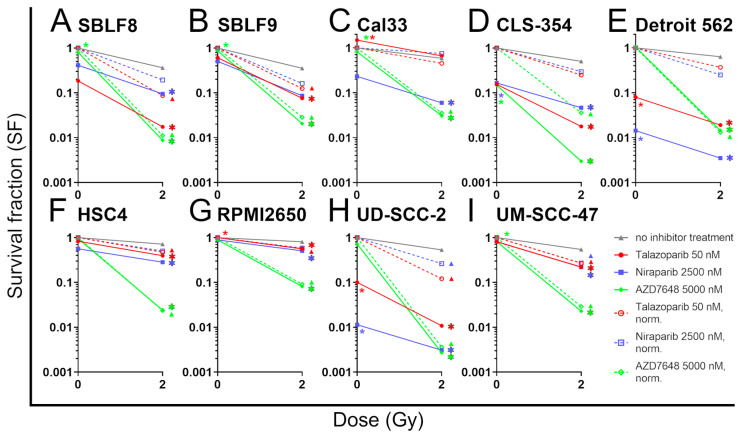
Colony formation assay. Survival fraction (SF) is displayed. Healthy fibroblast cells (**A**) SBLF8, (**B**) SBLF9, HPV-negative HNSCC cells (**C**) Cal33, (**D**) CLS-354, (**E**) Detroit 562, (**F**) HSC4, (**G**) RPMI2650 and HPV-positive HNSCC cells (**H**) UD-SCC-2, (**I**) UM-SCC-47 were treated either with talazoparib (50 nM), niraparib (2500 nM) or AZD7648 (5000 nM) or combined with 2 Gy IR. After treatment, solitary cells were allowed to form colonies for 10–14 days. Colonies containing at least 50 cells were counted. Inhibitors are displayed in color according to the legend. Solid lines display the data of control and KI with and without IR. Dashed lines represent a normalization, where the effect of KI alone is eliminated, and the difference between the normalized KI + IR and IR alone represents supra-additivity. Each value represents mean ± SD (*n* ≥ 4, two technical replicates each). Compared to IR alone, the significance of combined treatments is represented by * on the right and supra-additivity by a triangle on the right. On the left, * represents a significant difference of KI alone compared to IR alone. When * is above the line, the effect of KI alone is weaker than that of IR alone and when * is below the line, the effect of KI alone is stronger. Significance was determined using a two-tailed Mann–Whitney U test with * *p* ≤ 0.05.

To assess the long-term ability of cells to proliferate after treatment, the colony formation assay is a crucial tool. Mono-treatment with both PARPis was toxic in most cell lines. This included normal tissue cell lines. There was no toxicity in the RPMI2650 and UM-SCC-47 cell lines, and talazoparib did not reduce the survival fraction in Cal33 and HSC4 (Figure 4A–I). At the same time, there was only a small effect of AZD7648 mono-treatment, including both healthy fibroblasts cell lines, with an SF of 0.83 in SBLF8 and an SF of 0.85 in SBLF9. The lowest survival rate in tumor cell lines was in the UD-SCC-2, at SF 0.79 (median). The only exception was CLS-354, which was already very responsive. IR alone was effective in all cell lines and was most effective in both the fibroblasts cell lines, with an SF of 0.34 in SBLF8 and an SF of 0.30 in SBLF9. Among the cancer cell lines, the SF was lowest in the cell line CLS-354 with 0.50. All three combined treatments of KI plus IR reduced the survival fraction compared to the IR mono-treatment in almost all cell lines. The only exceptions are niraparib plus IR in SBLF9 and talazoparib plus IR in Cal33. This also means that the combined treatment with AZD7648 plus IR resulted in a significant decrease in the survival fraction in all cell lines. Talazoparib had supra-additive and, therefore, radiosensitizing effects in SBLF8 and SBLF9 and the cancer cell lines, while sparing Cal33, CLS-354 and Detroit 562. Niraparib radiosensitized the two HPV-positive cell lines, UD-SCC-2 and UM-SCC-47. Further data on supra-additive effects on cell inactivation in the colony formation assay is provided in Supplementary Table S3.

Out of the three KIs investigated, AZD7648 plus IR had the most potent radiosensitizing effect in each cell line, with normalized survival fractions ranging from 0.05 in RPMI2650 to 0.002 in UD-SCC-2. This effect was supra-additive in all cell lines. It is important to note that this includes the healthy fibroblast cell lines SBLF8 and SBLF9.

**Figure 5:**
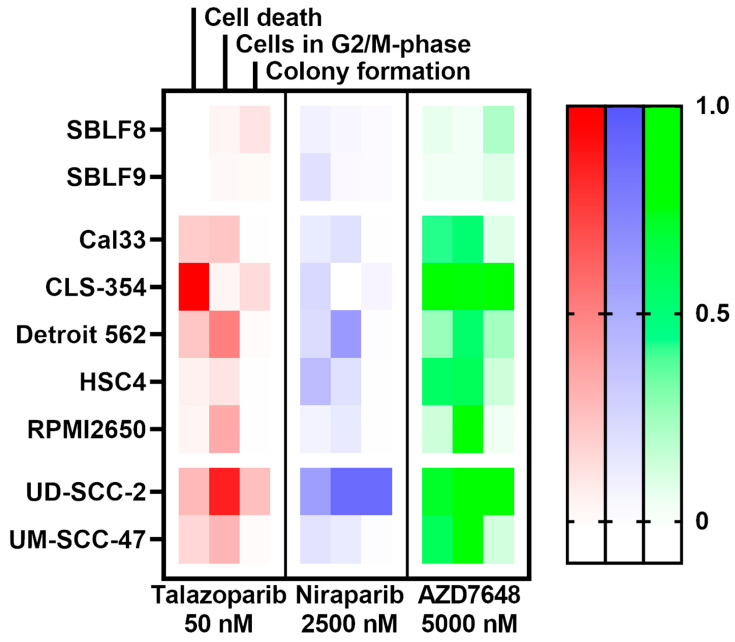
Overview of all effect sizes of combined treatments compared to IR alone. All combined treatments across all methods and all cell lines are aggregated in this heat map. Values were calculated using the mean values either by subtracting the effect of IR alone from the effect of combined treatment (cell death; cells in G2/M-phase) or by dividing the SF of IR alone by the SF of the combined treatment (colony formation). The highest resulting value within one method, but across all inhibitors and cell lines, was normalized to the value “1”. Inhibitors are color-coded, and color intensity correlates with the normalized value for the effect of combined treatment compared to IR alone.

A visual overview of the efficacy of each combined treatment is displayed as a heat map, covering all methods and cell lines (Figure 5). The higher color intensity represents a stronger effect of the combined treatment compared to IR alone. The combined treatments affected the two fibroblast cell lines significantly less compared to the HNSCC cell lines. The HPV-positive cell lines, but especially UD-SCC-2, seem to be very reactive to all the combined treatments, and AZD7648 stands out as more consistently effective compared to talazoparib and niraparib.

## 3. Discussion

We aimed to assess if talazoparib, niraparib and AZD7648 radiosensitize HNSCC cell lines in vitro, and whether they are less toxic for healthy fibroblast cells and should therefore be further considered for clinical testing in HNSCC. While most of the comparable studies focus on clonogenic survival alone, we also measured apoptotic and necrotic cell death and G2/M-phase arrest, in order to get a more comprehensive picture of the effects resulting from the combined treatment with KI plus IR.

Unrepairable DNA damage leads to the activation of various cellular processes. For example, it can lead to cell death through apoptosis or necrosis induction or inhibit proliferation through senescence, where a cell grows in size and loses its ability to proliferate [71,72]. Tumor cells survive because these mechanisms are compromised in them [27]. Therefore, finding a treatment that induces cell death or senescence in tumor cells is desirable. We directly measured apoptotic and necrotic cell death induction and found AZD7648 plus IR to increase cell death in all HNSCC cell lines, except RPMI2650, which did not respond with a significant increase in cell death to any treatment. These effects were mostly supra-additive. Talazoparib and niraparib with IR prompted rather heterogeneous effects, being effective in only five and four of seven HNSCC cell lines, respectively. In the healthy fibroblast cell lines, the combined treatments did not relevantly increase cell death.

Next, the G2/M-phase arrest is of interest because G2/M is the most radiosensitive phase of the cell cycle [69,70]. In a clinical setting with fractionated radiotherapy, a patient receives radiotherapy every 24 h. If a treatment results in an increased G2/M-phase arrest for at least 48 h, the next cycle of IR may target many cells that are more vulnerable to irradiation, resulting in further increased DNA damage. Contrary to tumor cells, healthy cells still have a functioning and effective G1 block. The G1 phase is much more radioresistant [69,70]. In theory, this circumstance should facilitate the tumor-selectivity of the treatment. Such an identified mechanism of tumor-selectivity is demanded by a 2018 report, claiming that initiating clinical trials without it was ill-advised [73]. To gain information on that, we measured the cell cycle distribution. As with cell death induction, AZD7648 had the strongest effects overall, radiosensitizing all HNSCC cell lines in a supra-additive manner, while talazoparib and niraparib did not do so consistently and did not have any effect in CLS-354. The combined treatments did not increase the G2/M-phase arrest in healthy fibroblasts. AZD7648 especially had no such effect on healthy fibroblasts at all.

The colony formation assay is the gold standard for evaluating in vitro radiosensitivity. It displays the ability of the cells for long-term proliferation after treatment. We found that all combined treatments were effective in suppressing colony formation, with AZD7648 being by far the most effective overall, with supra-additive effects in all cell lines. The combined treatments also had relevant effects in the two healthy fibroblast cell lines, with AZD7648 being the most effective again. However, while talazoparib and niraparib alone were toxic, AZD7648 alone had very little effect on healthy fibroblasts.

Overall, we found all three KIs to be promising, with AZD7648 being the most potent, while irradiation-induced damage to healthy tissue is a concern. In the following, we first focus on the PARPis talazoparib and niraparib before moving on to the DNA-PKcsi AZD7648. Both are discussed, beginning with their effects on HNSCC cell lines and continuing with an evaluation of their effects in healthy fibroblasts.

### 3.1. Talazoparib and Niraparib Radiosensitize HNSCC Cell Lines Heterogeneously

PARP is involved in SSB repair. The inhibition of PARP combined with an inductor of DNA damage such as IR should therefore lead to the accumulation of SSBs, which can then transform into the much more toxic DSBs in the S-phase [14,17]. The PARPis talazoparib and niraparib are already market-approved in some gynecological tumor entities [42,43,44,45,46,47]. While multiple preclinical studies have found olaparib, another PARPi, to successfully radiosensitize HNSCC cell lines in vitro [48,51], data on the effects of talazoparib and niraparib combined with IR in HNSCC are scarce. The potential of a combined treatment with IR in HNSCC cell lines in vitro has been shown by one study for talazoparib [52] and by two studies for niraparib [49,50].

In our study, we are furthering this knowledge by examining additional cell lines, including HPV-negative and HPV-positive ones, as well as healthy fibroblasts, and by measuring cell death induction and G2/M-phase arrest, in addition to clonogenic survival. We found both talazoparib and niraparib to have radiosensitizing effects in HNSCC cell lines. Overall, neither PARPi had a clear advantage over the other. The HPV-positive cell lines seemed to be more consistently affected by the combination of PARPis plus IR compared to the HPV-negative cell lines, where the effects were more heterogeneous. This was particularly prominent in apoptosis and necrosis induction. The G2/M-phase arrest was detectable for the combined treatment with PARPis plus IR for all cell lines, except for the HPV-negative cell line CLS-354. While CLS-354 stands out in this way, it is important to note that the effects in the other cell lines were heterogeneous in size, and in HSC4, for example, the effects were very small. In clonogenic survival, almost all cell lines were affected by both combinations, however, in the HPV-positive cell lines, the effects were supra-additive, while the decrease in SF was additive in most HPV-negative ones. Further in vivo studies are warranted, and establishing a predictive marker for the effectiveness of PARPis plus IR in HNSCC would be desirable.

The higher radiosensitivity of the HPV-positive HNSCC has been known for a long time [9,11,12]. We show that the HPV-positive HNSCC is not only more radiosensitive per se, but also exhibits supra-additive effects when treated with talazoparib or niraparib in combination with IR. These effects represent radiosensitization capabilities, which were not consistently observed in the case in HPV-negative cell lines. Comparable data on this are conflicting, and the results depend on the experimental design and the specific inhibitor used. While the results of one study on talazoparib are similar to our findings [52], two studies on niraparib found a slightly stronger effect in HPV-negative cell lines [49,50]. However, these differences seem negligible since all the mentioned studies found effects in both HPV-negative and HPV-positive cell lines. It is worth mentioning that, at 50 nM, we used a lower concentration of talazoparib compared to the 100 nM used in the aforementioned study [52]. The two studies on niraparib showed these promising results with a concentration of 1000 nM [49,50], while we used 2500 nM. Despite that, the results are similar. Zhou et al. concluded that a combined treatment of PARPis and IR should be further considered in HNSCC, especially in the more radioresistant HPV-negative cell lines [52]. Our findings not only support but extend beyond their conclusion. All in all, in our experiments, despite the fact that the HPV-positive cell lines were overall more strongly affected, PARPis with IR affected all HNSCC cell lines, both HPV-negative and HPV-positive, in a relevant fashion.

Therefore, we conclude that for the potential treatment of HNSCC tumors, regardless of HPV status, the combination of IR with PARPi, specifically talazoparib and niraparib, should be considered for further studies. As the response to the combined treatment was to some extent heterogeneous between cell lines, individual testing prior to treatment with PARPis and IR seems reasonable. It would therefore be desirable to explore the underlying molecular mechanisms involved in the heterogeneity of the effects in order to find a predictive marker for therapeutic success.

One possible approach to understanding the heterogeneity in response to treatment with PARPis and IR, especially among the HPV-negative HNSCC cell lines, is the influence of p53. The p53 protein is a major component in the regulation of cellular responses to stress, including apoptosis, necrosis, cell cycle, cell proliferation, DNA repair and senescence [74,75]. While HPV-positive HNSCC cells usually encode a wild-type (wt) p53, which is then inactivated and degraded by the HPV oncoproteins E6, E7 and others, rendering the HPV-positive HNSCC functionally p53-deficient [76,77,78], the HPV-negative HNSCC can be divided into two groups, based on their p53 status: p53 wt and p53 mutated (mut). In a clinical sample of 243 patients with HPV-negative HNSCC, 84% were p53 mut [79]. The p53 status is of interest in the context of our study, since PARP is involved in p53 regulation [80,81]. On the one hand, there are studies that found a functional p53 to promote a radiosensitizing effect of PARPis in different tumor entities [82,83], while, on the other hand, there is evidence that the loss-of-function mutations of p53 lead to increased radiosensitivity by PARPis through the activation of oxidative stress pathways [84]. The accumulated evidence suggests that PARPis can have radiosensitizing effects in p53 wt, as well as in p53 mut tumors, through various different mechanisms [85].

Out of the five HPV-negative cell lines we investigated, Cal33, HSC4 and Detroit 562 are p53 mut [86,87], while RPMI2650 is a p53 wt [88]. The p53 status of CLS-354 is unclear, although one study found p53 expression in CLS-354 after treatment with IFNγ, which can trigger cell cycle arrest and apoptosis, among other functions [89]. In our data, no clear association can be made between the p53 status of an HPV-negative HNSCC cell line and the radiosensitization capabilities of either of the two PARPis. This is stressed by the fact that even within one cell line and method, oftentimes, the radiosensitizing effects of talazoparib and niraparib are incongruent. In our experiments, the influence of the p53 status of the HPV-negative HNSCC cell lines on the radiosensitizing capabilities of PARPis was not great enough to be noticeable among the effects of various other differences between the cell lines that may influence treatment effectiveness, and it does not explain the heterogeneity found.

### 3.2. Effects of Talazoparib and Niraparib on Healthy Fibroblast Cells

In addition to the therapeutically desirable effects of a treatment, it is important to consider the effects on healthy tissue as well. Since talazoparib and niraparib are already market-approved, their safety and tolerability have been proven before [90,91]. Nevertheless, contrary to the above-mentioned studies on PARPis plus IR in HNSCC [49,50,52], we included two healthy fibroblast cell lines that were treated and evaluated exactly the same as the HNSCC cell lines for comparison. We focused on how the PARPis could amplify the potential irradiation-induced side effects.

On the one hand, neither combination of PARPi and IR increased cell death or G2/M-phase arrest compared to IR alone in SBLF8 or SBLF9. On the other hand, in the colony formation assay, both combined treatments showed toxicity, resulting in a decreased SF compared to IR alone, for talazoparib in both fibroblast cell lines and for niraparib in SBLF8. This constellation leads us to hypothesize that the irradiation-associated effects of talazoparib and niraparib on healthy fibroblasts may be mediated by the induction of senescence. The possible induction of senescence by KIs with IR and the implications for clinical development are discussed in more detail below in the context of the DNA-PKcsi AZD7648.

Additionally, the high G2/M-phase arrest by mono-treatment with talazoparib and especially niraparib in SBLF8 is noteworthy. Interestingly, this effect vanished in combination with IR. We do not have a conclusive explanation for this unusual behavior and attribute it to individual cellular composition of the donor of the fibroblast cell line. Unfortunately, we cannot investigate the context of this phenomenon further due to the protection of the donor’s privacy.

To summarize, our experiments suggest that increased irradiation-induced side effects are likely but the anti-tumor potential of talazoparib and niraparib with IR justifies further consideration of these drugs for treatment of HNSCC.

### 3.3. AZD7648 Is a Potent Radiosensitizer in HNSCC Cell Lines

AZD7648 is a potent and selective inhibitor of the DNA-PKcs with radiosensitizing capabilities [92] first discovered in 2020 [93]. In HR-deficient tumor cells, inhibition of NHEJ through DNA-PKcsis combined with DNA damage inductors like IR leads to accumulation of DSBs in the tumor cells which results in cell death [14,15,16]. Meanwhile, healthy cells are still HR-proficient, which should facilitate tumor-selectivity.

We found AZD7648 to be a highly effective radiosensitizer of HNSCC cell lines in vitro, more effective than the PARPis talazoparib and niraparib. The combination of AZD7648 and IR induced cell death in all the HNSCC cell lines but RPMI2650, which was resistant to all the combined treatments. For the induction of G2/M-phase arrest and for clonogenic survival, the picture gets even clearer. AZD7648 plus IR stood out as by far the most powerful radiosensitizer, having supra-additive effects in all cell lines, irrespective of HPV status. Our data support the idea that AZD7648 has the potential to relevantly improve radiation therapy in HNSCC. Because AZD7648 has only recently been discovered, not much data exist on HNSCC. One study was conducted, which found AZD7648 to be a potent radiosensitizer, additionally, in two HNSCC cell lines in a mouse model, in both oxic and anoxic conditions [64]. Another study found promising effects of AZD7648 plus IR in vitro, especially in the two HPV-negative HNSCC cell lines investigated [65].

DNA-PK inhibitors (DNA-PKi) other than AZD7648 have been found to radiosensitize HNSCC cell lines. Comparable to our results, DNA-PKi KU57788 and IC87361 did so in a more potent way than the PARPis olaparib and veliparib [61]. Studies on other DNA-PKi, and also inhibitors of ATM, ATR and PARP, did not find any therapeutically exploitable differences between photon and proton irradiation in HNSCC cell lines [94,95]. Additionally, the combination of DNA-PKis with other substances can amplify the radiosensitizing effects in HNSCC. This has been shown for a combination with ATR inhibition [62], as well as with the PARPi olaparib, where the effect was comparable to cisplatin plus IR, while having considerably fewer toxic effects [63]. However, the effects of AZD7648 alone on healthy cells should be considered first, before proposing its combination with another radiosensitizer to enhance the radiosensitizing effects.

### 3.4. Healthy Fibroblast Cells Tolerate AZD7648 Well While Combining AZD7648 with IR Impairs Clonogenicity without Increased G2/M Arrest, Apoptosis or Necrosis

The high radiosensitization capability of AZD7648 in HNSCC cell lines begs the question to what degree healthy tissue is affected by it as well. We found that AZD7648 alone was tolerated very well in healthy fibroblasts despite the relatively high concentration of 5000 nM. There was no increase in the G2/M-phase arrest compared to the control, while one of the two healthy fibroblast cell lines experienced a slight increase in cell death. Clonogenicity was only marginally affected in SBLF8 (SF = 0.83) and not affected in SBLF9 (SF = 0.85) compared to the control which was normalized to the value of 1. For comparison, SF for IR alone was 0.34 and 0.30, respectively.

Next to the tolerance of the substance alone, potential effects in the irradiated area are a concern. One study found AZD7648 to relevantly radiosensitize intestinal and mucosal stem cells in mouse models [64]. The effects of AZD7648 plus IR in healthy fibroblast cell lines observed in another study are very similar to our observations [65]. In our analyses, AZD7648 had no radiosensitizing effects on healthy fibroblasts regarding cell death induction and G2/M-phase arrest. However, we could observe a strong decrease in the survival fraction compared to the IR alone. This leads us to hypothesize that AZD7648 with IR induces senescence in healthy fibroblast cells. Noticeably, the data on the dual DNA-PK/mTOR inhibitor CC-115 in similar fibroblast cell lines, where senescence was directly measured, do not support this hypothesis, since the CC-115 with IR decreased senescence compared to the IR alone, rather than inducing it [29]. Not having measured senescence directly but only indirectly, through clonogenic survival, is a clear limitation of our study. To test the hypothesis of senescence induction in healthy fibroblasts after treatment with AZD7648 and IR, we propose that further studies directly measure senescence after treatment using beta-galactosidase.

When healthy tissue experiences senescence, the senescent cells maintain tissue integrity. In contrast, after cell death, tissue integrity is lost. Side effects caused by induction of senescence in healthy tissue are likely less burdening for patients than those mediated by direct cell death and, therefore, cell loss, for example through apoptosis or necrosis [96]. Nevertheless, the irradiation-induced side effects of AZD7648 remain a concern and can only be adequately evaluated in clinical trials. There is one currently in progress, which evaluates AZD7648 alone and in combination with other anti-cancer agents, but not including IR (NCT03907969).

It is an obvious limitation of an in vitro study like this one that the results cannot be extrapolated into more complex systems, such as in vivo models, or into a clinical setting. There are a lot of factors that influence how the treatment affects the tumor cells in such settings, such as the distribution of the inhibitor, the tumor microenvironment and the immune system. Additional studies are therefore essential, and we believe that further in vivo studies and, as a consequence of these studies, potentially clinical trials are warranted given the high potential of AZD7648 to radiosensitize HNSCC cell lines. These studies can address the complexity of the tumor microenvironment in vivo and systemic effects that cannot be assessed in vitro.

Additionally, further in vitro analyses that could shed light on ways to avoid normal tissue damage include combining PARPis and DNA-PKcsis, while reducing the respective concentrations, as well as lowering the dose of ionizing radiation. Assessing cell death and cell cycle arrest at multiple time points after treatment to see differences in the response over time can also be of interest. Replicating and validating our results in organoids would be particularly compelling.

## 4. Materials and Methods

### 4.1. Cell Lines and Cell Culture

This study examined two healthy fibroblast cell lines, SBLF8 and SBLF9, and seven HNSCC cell lines. SBLF8 and SBLF9 were derived from healthy donors, while Cal33 (CVCL_1108), HSC4 (CVCL_1289), UD-SCC-2 (CVCL_E325) and UM-SCC-47 (CVCL_7759) were obtained from Dr. Thorsten Rieckmann (University of Medical Centre Hamburg-Eppendorf, Germany); CLS-354 (Cytion Catalog Number 300152), Detroit 562 (300399) and RPMI2650 (300323) were obtained from Cytion (formerly CLS, Eppelheim, Germany). UD-SCC-2 and UM-SCC-47 are HPV-positive, while the remaining five HNSCC cell lines are HPV-negative. Cal33, HSC4 and UM-SCC-47 were derived from tumors of the oral tongue, and CLS-354 from a tumor of the oral cavity. RPMI2650, Detroit 562 and UD-SCC-2 were derived from tumors of the nasal septum, the pharynx and the hypopharynx, respectively. All cells were incubated in a humidified environment at 37 °C and 5% CO_2_ and kept in cell culture flasks. The medium for SBLF8 and SBLF9 consisted of an F-12 medium supplemented with 15% fetal bovine serum (FBS, Sigma-Aldrich, St. Louis, MO, USA), 2% non-essential amino acids (NEA, Gibco, Waltham, MA, USA) and 1% penicillin-streptomycin (Gibco, Waltham, MA, USA), while HNSCC cells were kept in Dulbecco’s Modified Eagle Medium (DMEM, Gibco, Waltham, MA, USA), supplemented with 10% FBS and 1% penicillin–streptomycin. Cells were passaged twice a week, or when they reached a confluence of 90%. The medium was removed, cells were washed with phosphate-buffered saline (PBS, Gibco, Waltham, MA, USA), trypsinated (Gibco, Waltham, MA, USA) and incubated for 2 to 5 min until they were detached from the flask. The trypsination was terminated with a fresh medium. The average number of passages the cell lines experienced before seeding was between 5 and 23 for all cell lines, except for Detroit 562 with 44, which had already been exposed to a high passage number before storage.

### 4.2. Kinase Inhibitors and Radiation Treatment

Every analysis consisted of eight settings per cell line. We analyzed three inhibitors, and each substance was evaluated alone and with 2 Gy IR. The remaining two settings contained dimethyl sulfoxide (DMSO, Roth, Karlsruhe, Germany) to compensate for its use as the carrier medium for the inhibitors, so that every setting contained identical volumes of DMSO. The setting treated only with DMSO will be referred to as control, and the setting treated with DMSO plus 2 Gy will be referred to as 2 Gy or IR alone. The radiation dose of 2 Gy was chosen, since it is well established in research and is a standard single dose in clinical practice. It is high enough to induce measurable effects, but low enough for a sufficient number of cells to survive the treatment with IR alone.

The inhibitors used were the PARPis talazoparib (50 nM) and niraparib (2500 nM) and the DNA-PKcsi AZD7648 (5000 nM), all obtained from Selleck Chemicals (Houston, TX, USA). The concentrations used for talazoparib and niraparib were determined by a calculation based on the maximum plasma concentration and the molecular weight by Jonuscheit et al. [97]. The concentration for AZD7648 was based on internal data by Klieber et al. [65]. For radiation treatment, an ISOVOLT Titan X-ray generator (GE, Ahrensburg, Germany) was used. It was operated at 120 kV and a dose rate of 6 Gy per minute.

### 4.3. Flow Cytometry Analysis of Apoptosis and Necrosis

Apoptosis and necrosis were assessed using flow cytometry. Cells were seeded in T25 flasks. After 24 to 96 h of incubation, when a confluence of about 80% was reached, the medium was changed to a medium with a reduced content of 2% FBS, and the inhibitor was added. After 3 h of incubation post-treatment, half of the flasks were irradiated with 2 Gy. After 48 h, cells and supernatants were harvested using trypsin and PBS. Centrifuge tubes containing 14 mL of cell suspension were centrifuged at 180× *g* for 8 min at room-temperature, the supernatant was disposed, and 300 µL cell solution remained. An amount of 150 µL was transferred into Eppendorf tubes.

An amount of 1 mL starvation medium (2% FBS) and 10 mL 70% ethanol (Fischer, Saarbruecken, Germany) were added to the centrifuge tubes containing the remaining 150 µL of cell suspension, which were then stored at 4 °C for a minimum of 24 h to be used for cell cycle analysis.

For apoptosis/necrosis analysis, 200 µL of cold Ringer´s solution (Fresenius, Bad Homburg, Germany), as well as 10 µL of an equal mixture of APC Annexin V (BD Biosciences, Franklin Lakes, NJ, USA) and 7AAD (BD Biosciences, Franklin Lakes, NJ, USA), was added to 150 µL cell suspension. After 30 min of incubation on ice without light exposure, the tubes were centrifuged (180× *g*, 8 min, RT), the supernatant was removed, and cells were resuspended. Another 150 µL of cold Ringer’s solution was added, and 200 µL of each tube was transferred onto a 96-well plate. A CytoFLEX S flow cytometer (Beckmann Coulter, Brea, CA, USA) was used for measurements. Data were analyzed with Kaluza Analysis software (Version 2.1, Beckmann Coulter, Brea, CA, USA). Representative gating strategies are displayed in Supplementary Figure S1.

Annexin V and 7AAD double negative (Ann7AAD − −) were defined as living, double positive (Ann7AAD + +) was defined as necrotic and Annexin V positive only (Ann7AAD + −) as apoptotic.

### 4.4. Flow Cytometry Analysis of Cell Cycle

Cell cycle analysis was conducted using flow cytometry. Seeding and harvesting were performed as described for apoptosis–necrosis analysis. The centrifuge tubes with cells fixed in ethanol were centrifuged (180× *g*, 8 min, RT), supernatants were removed, and the pellet was resuspended. An amount of 1 mL of cold Ringer´s solution and 3 µL of 10-fold soluted Hoechst 33342 (Molecular Probes, Eugene, OR, USA) were added per sample, after which they were incubated on ice for 1 h. After another centrifugation cycle, disposing of the supernatant and resuspending, 150 µL of cold Ringer´s solution was added, resulting in a total of about 200–250 µL of cell suspension. For each condition, 200 µL of the resulting cell suspension was transferred into a 96-well plate.

CytoFLEX S flow cytometer (Beckmann Coulter, Brea, CA, USA) was used for measurements. Data were analyzed with Kaluza Analysis software (Version 2.1, Beckmann Coulter, Brea, CA, USA). Representative gating strategies are displayed in Supplementary Figure S1. Gating excluded doublets, and G1/G0-, S- and G2M-phase were differentiated by the intensity of the Hoechst signal.

### 4.5. Colony Formation Assay

Following 48 h of passaging, when confluence equated to about 80%, cells were harvested and seeded into Petri dishes. Depending on cell line growth characteristics and intended treatment, between 250–1000 cells were added to 3 mL of cell line-specific medium. They were then evenly distributed throughout the Petri dish by swirling the dish so that cells were separated by a sufficient distance. Cells were incubated overnight, but not longer than for a maximum of 24 h, until they were attached to the bottom of the Petri dish, but before they had the opportunity to proliferate, as this would have corrupted the statistical analysis. If a single cell had enough time to proliferate, the probability of this aggregate forming a colony would be disproportionately higher than that for cells that did not proliferate, since both cells would have to be damaged for them not to form a colony. This can be avoided by limiting the incubation time to a maximum of 24 h or less, which is usually not enough time for a relevant number of individual cells to divide. Then, the inhibitor was added, and 3 h later, half of the samples were irradiated.

After another 48 h of incubation, the medium was replaced, and cells were then incubated for the next 10–14 d, depending on their growth rate. The colony formation process was terminated by disposing of the medium and adding Wright’s eosin methylene blue solution (Carl Roth, Karlsruhe, Germany) until the bottom of the Petri dish was completely covered. After 30 min, the methylene blue staining was removed, and the Petri dishes were washed with water and deionized water. All samples were allowed to dry overnight, and images were captured using a stationary camera under specific lighting conditions. Colony counting was performed by using the images of the Petri dishes, and only those colonies with at least 50 cells were counted. Representative Petri dish images are displayed in Supplementary Figure S2. Plating efficiency (PE) describes the fraction of seeded cells able to form a colony in the control:

PE = colonies [control]/seeded cells [control].(1)

Survival fraction (SF) is calculated after treatment using the PE calculated from the corresponding control:

SF = colonies [treatment]/(seeded cells [treatment] × PE).(2)

### 4.6. Statistical Analysis

GraphPad Prism 9 (GraphPad Software, Boston, MA, USA) was used for statistical analysis. Each experiment was repeated at least four times to allow for statistical analysis using an unpaired, two-tailed Mann–Whitney U-test. A *p*-value of 0.05 determined statistical significance. A minimum of four replicates with two technical replicates were performed in the colony formation assay.

To study radiosensitization, supra-additivity was assessed. We added the effects of mono-treatment with IR and mono-treatment with an inhibitor together, and compared the additive effect to the effect of the combined treatment. Supra-additivity means that the effect of the combined treatment was greater than the added isolated effects. This means that there could be a clinical advantage of the combined treatment because there is a synergistic effect that extends beyond the added effects. Such supra-additivity points to the fact that the inhibitor is capable of radiosensitizing the cell line, rather than just having a toxic effect that adds to the effect of the IR. Therefore, we show if there is a radiosensitizing mechanism involved through the concept of supra-additivity.

Supra-additivity was assessed using the aforementioned unpaired, two-tailed Mann–Whitney U-test, where, again, a *p*-value of 0.05 determined statistical significance. For induction of apoptosis and necrosis and of G2/M-phase arrest using values in percent, the comparison was between

[IR] − [Co] + [KI] − [Co] vs. [KI+IR] − [Co].(3)

For colony formation assay, SF was used, and the comparison was between

[IR] vs. [KI + IR] / [KI].(4)

In each case, the effect of the corresponding approach is shown in square brackets, where Co = control without treatment, IR = ionizing radiation, KI = kinase inhibitor.

## 5. Conclusions

In conclusion, we found that the PARPis talazoparib and niraparib and the DNA-PKcsi AZD7648 relevantly radiosensitize HNSCC cell lines in vitro, with AZD7648 being the most potent and effective across all cell lines. Talazoparib and niraparib in combination with IR resulted in more heterogeneous effects, depending on the cell line. Therefore, it appears that for PARPis, individual patient testing would be necessary. AZD7648 alone had little to no effect on healthy fibroblast cells, making toxicity unlikely. The combination of AZD7648 with IR did not relevantly affect cell death or G2/M arrest in healthy fibroblast cells, but did decrease clonogenic survival relevantly, rendering radiation-induced side effects a concern. In summary, while talazoparib and niraparib are promising as well, AZD7648, especially, has the potential to improve radiation therapy in HNSCC in the future. Our findings justify in vivo studies, especially on the DNA-PKcsi AZD7648 combined with IR in HNSCC.

## Data Availability

The data used and analyzed during the current study are available from the corresponding author on reasonable request.

## Supplementary Materials

The following supporting information can be downloaded at: https://www.mdpi.com/article/10.3390/ijms25115629/s1.
