# Supplementary material for: Effective Radiosensitization of HNSCC Cell Lines by DNA-PKcs Inhibitor AZD7648 and PARP Inhibitors Talazoparib and Niraparib
Source: Int J Mol Sci. 2024 May 22;25(11):5629. doi: 10.3390/ijms25115629 (PMC11172136; doi:10.3390/ijms25115629)

Supplementary Figure S1: Representative Gating Strategies in Flow Cytometric Measurements

Effective Radiosensitization of HNSCC cell lines by DNA-PKcs Inhibitor AZD7648 and PARP Inhibitors Talazoparib and Niraparib

Displayed are representative gating strategies for flowcytometric measurements of apoptosis- and necrosis-induction and G2/M phase arrest-induction for SBLF8 and Cal33, representative of healthy fibroblast cell lines and HNSCC cell lines for all treatments.

Induction of apoptosis and necrosis in SBLF8

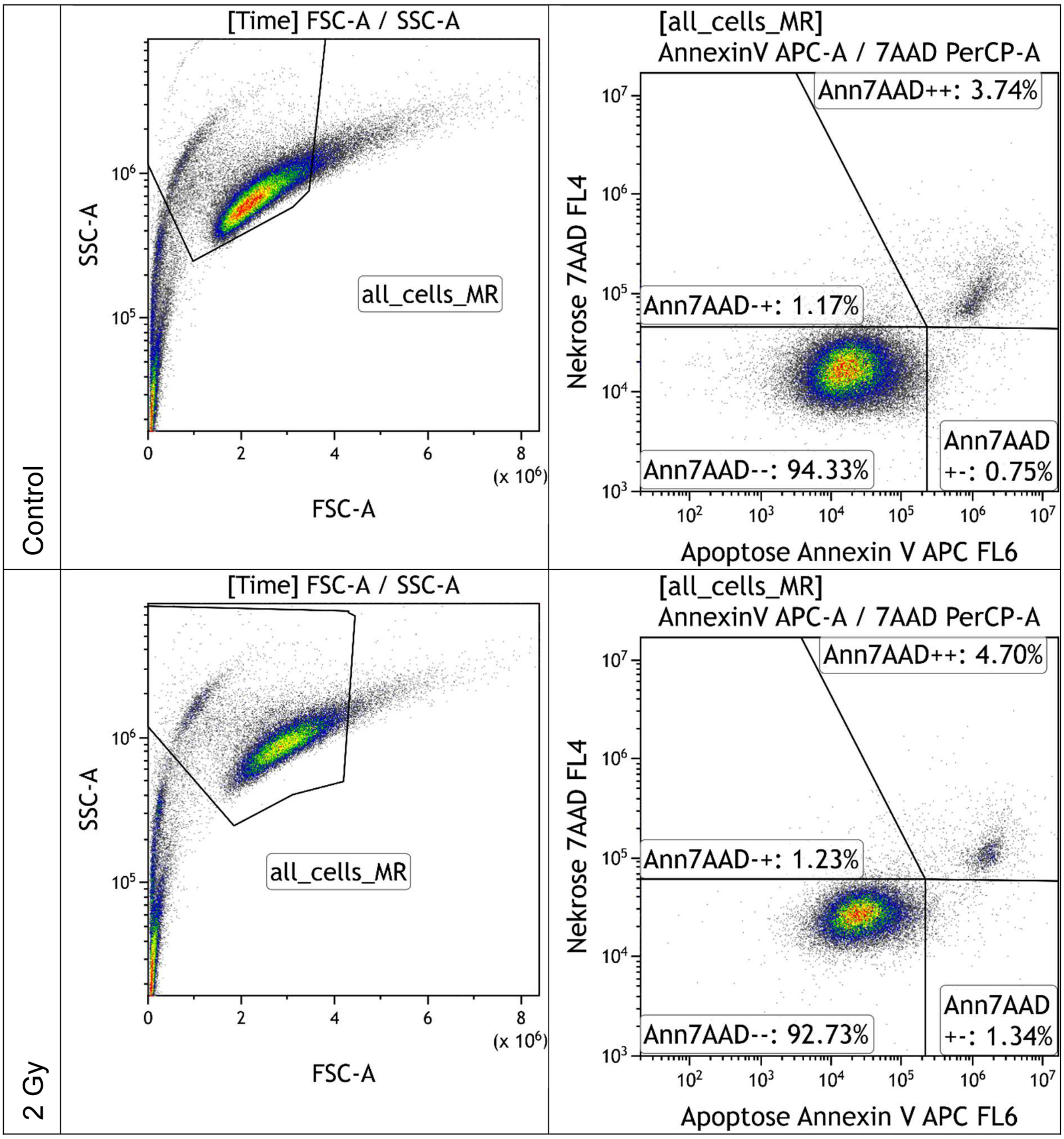

Induction of apoptosis and necrosis in SBLF8

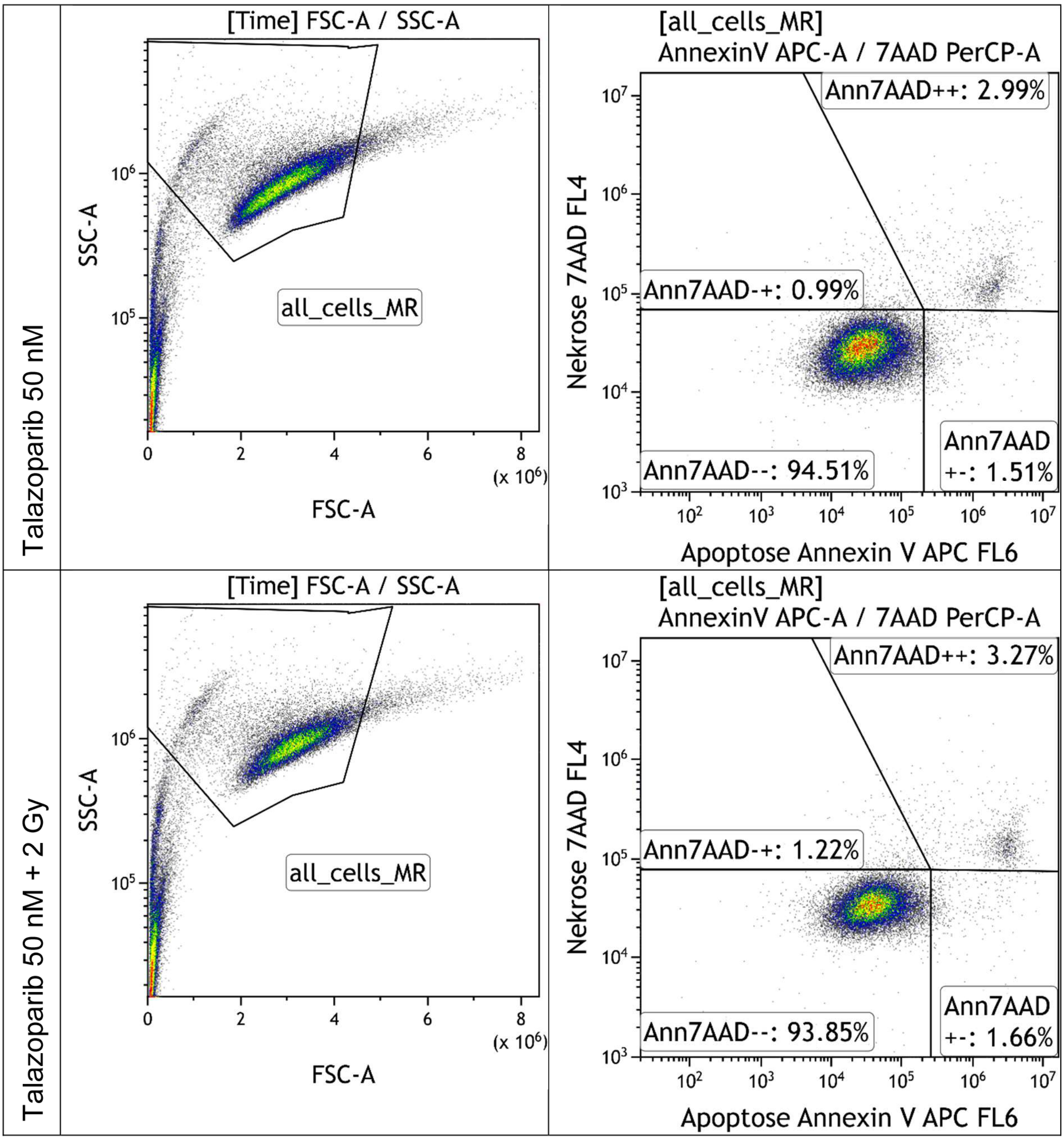

Induction of apoptosis and necrosis in SBLF8

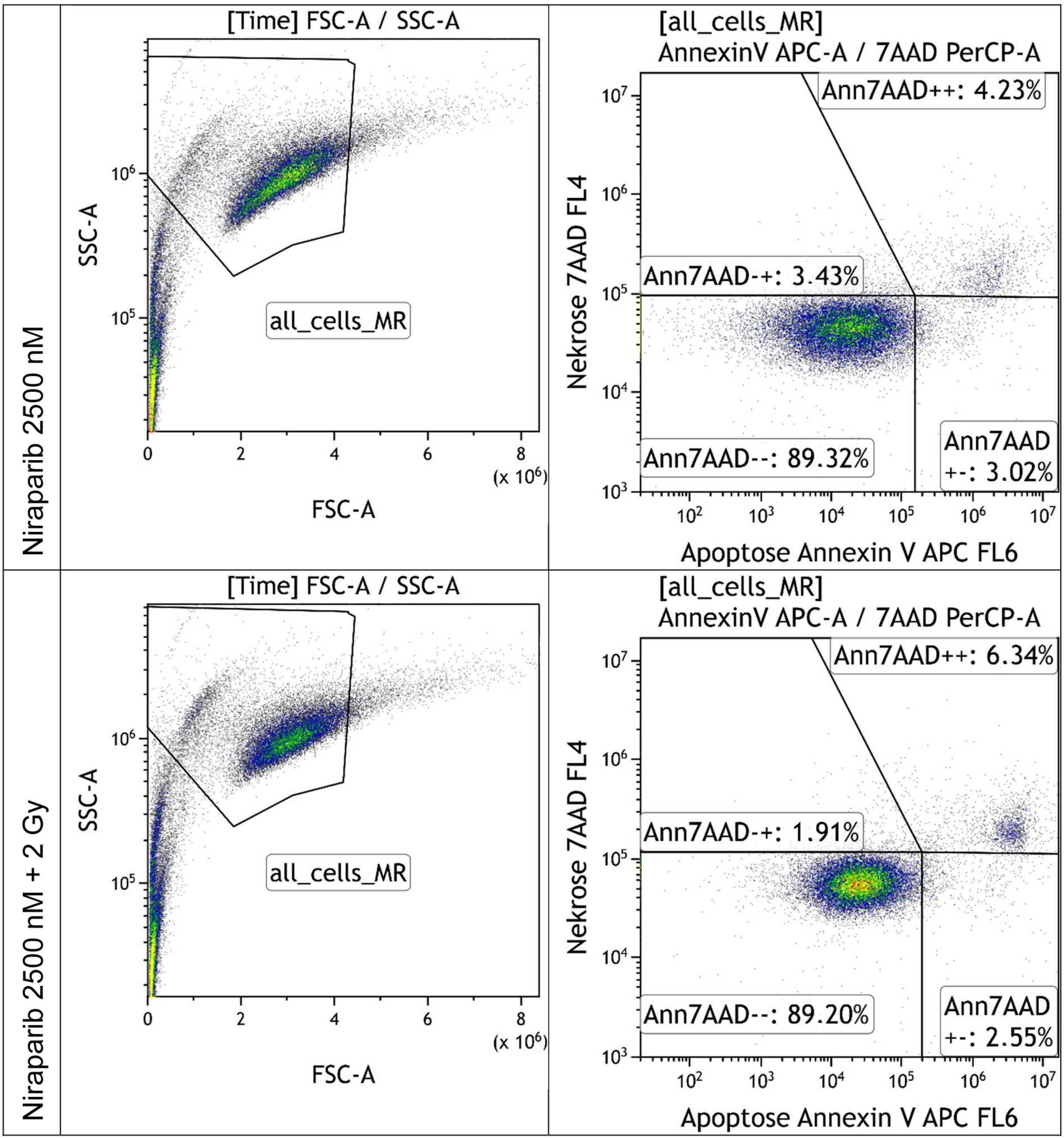

Induction of apoptosis and necrosis in SBLF8

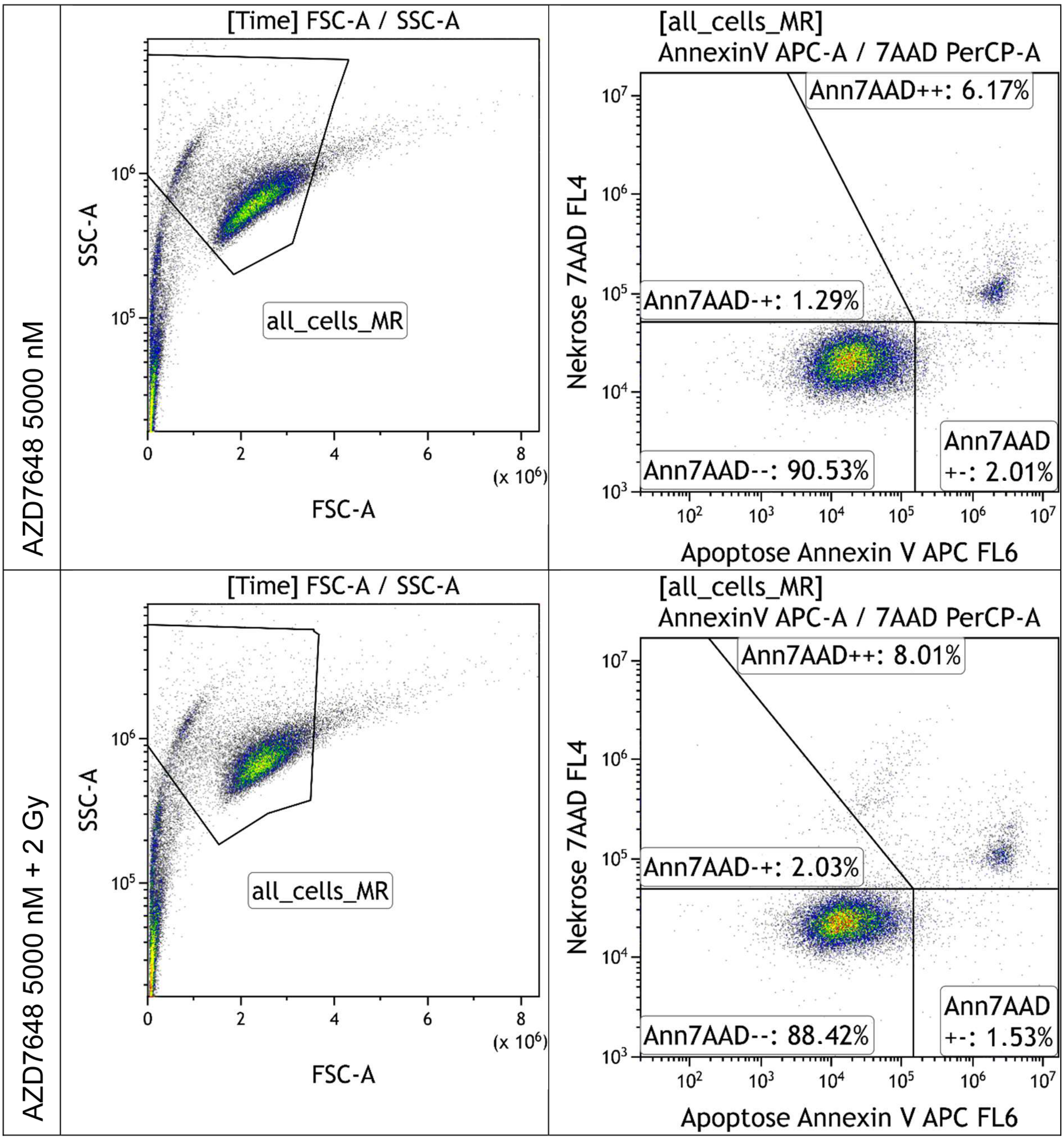

Induction of apoptosis and necrosis in Cal33

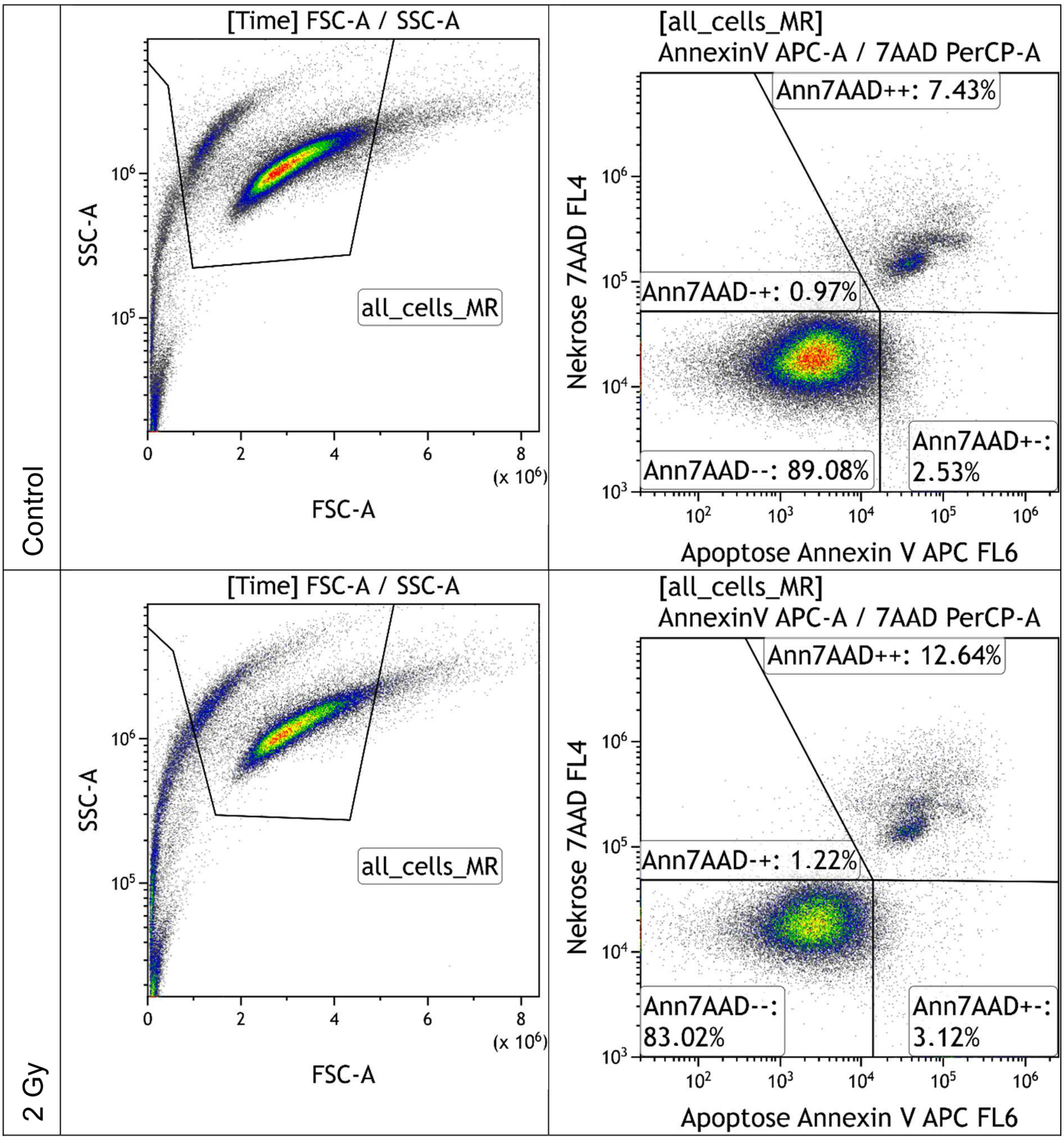

Induction of apoptosis and necrosis in Cal33

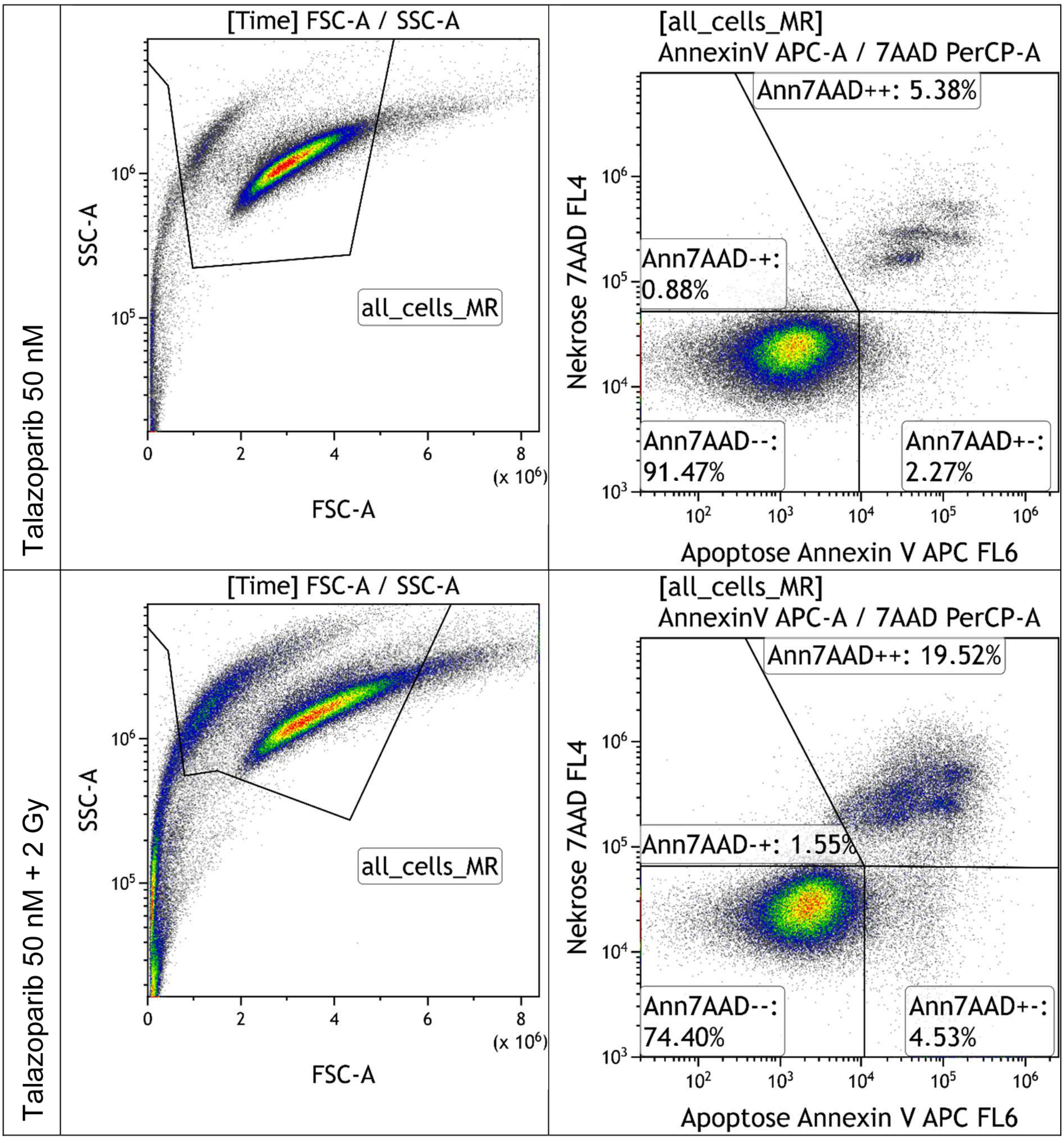

Induction of apoptosis and necrosis in Cal33

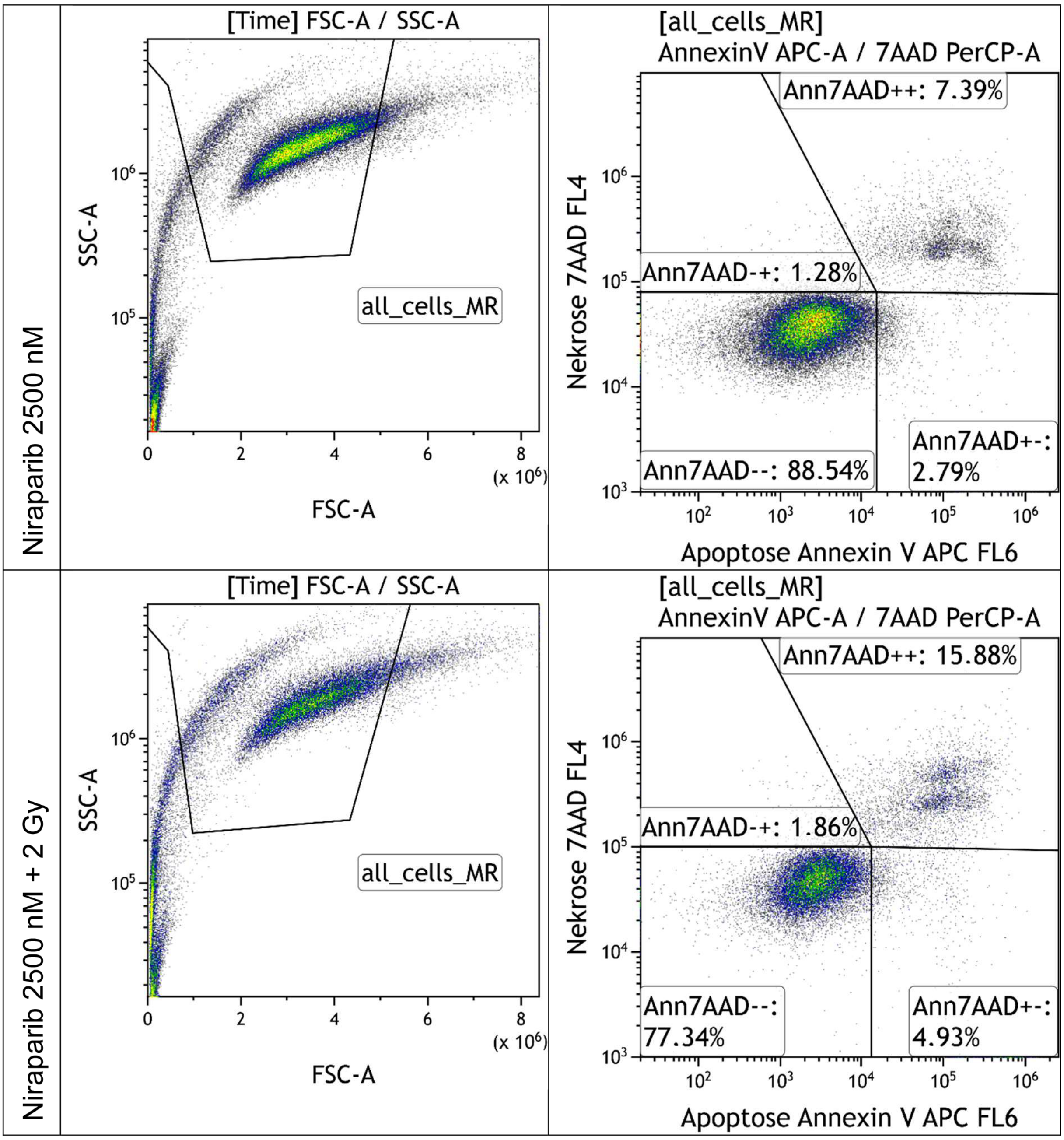

Induction of apoptosis and necrosis in Cal33

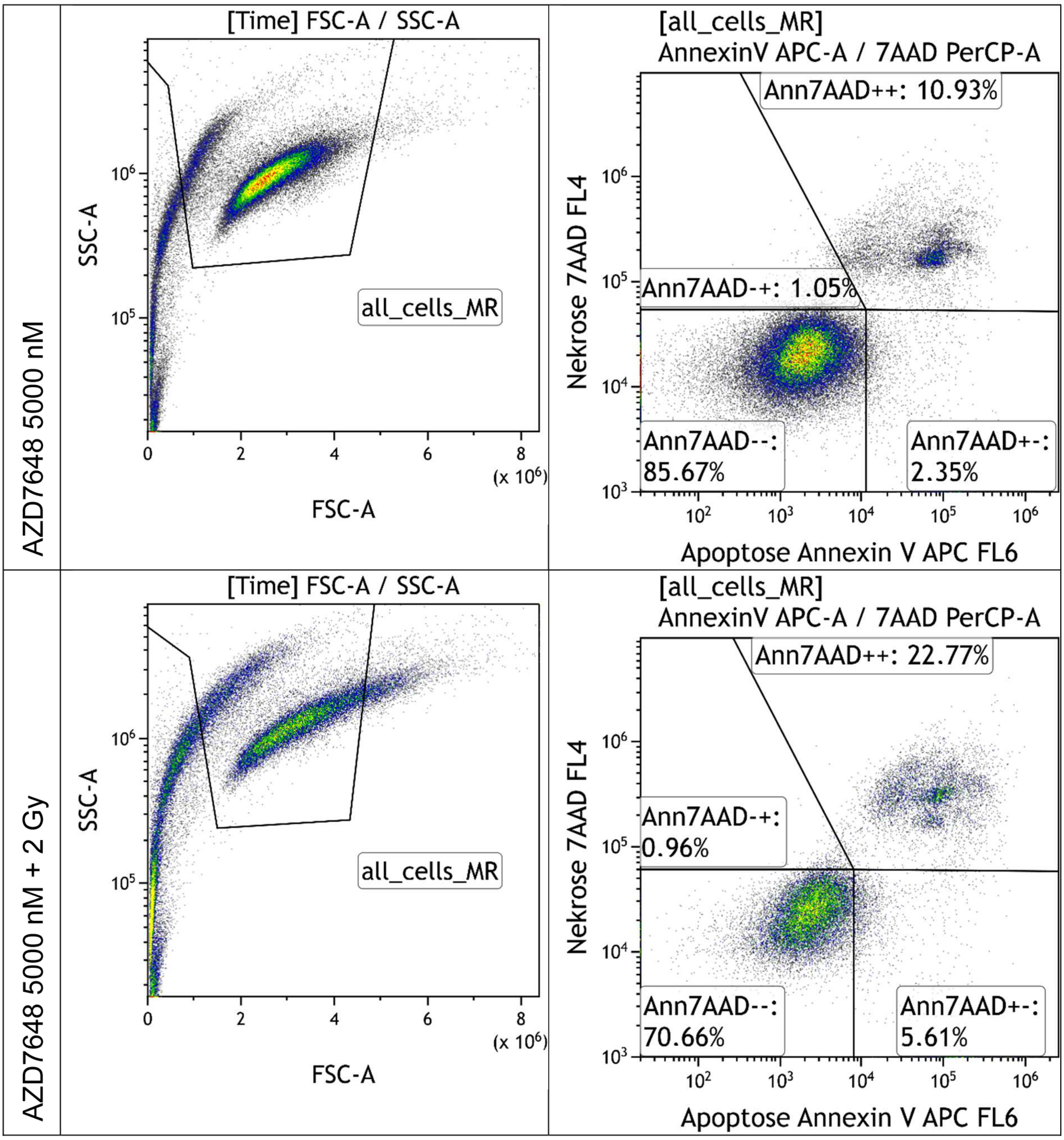

Induction of G2/M phase arrest in SBLF8

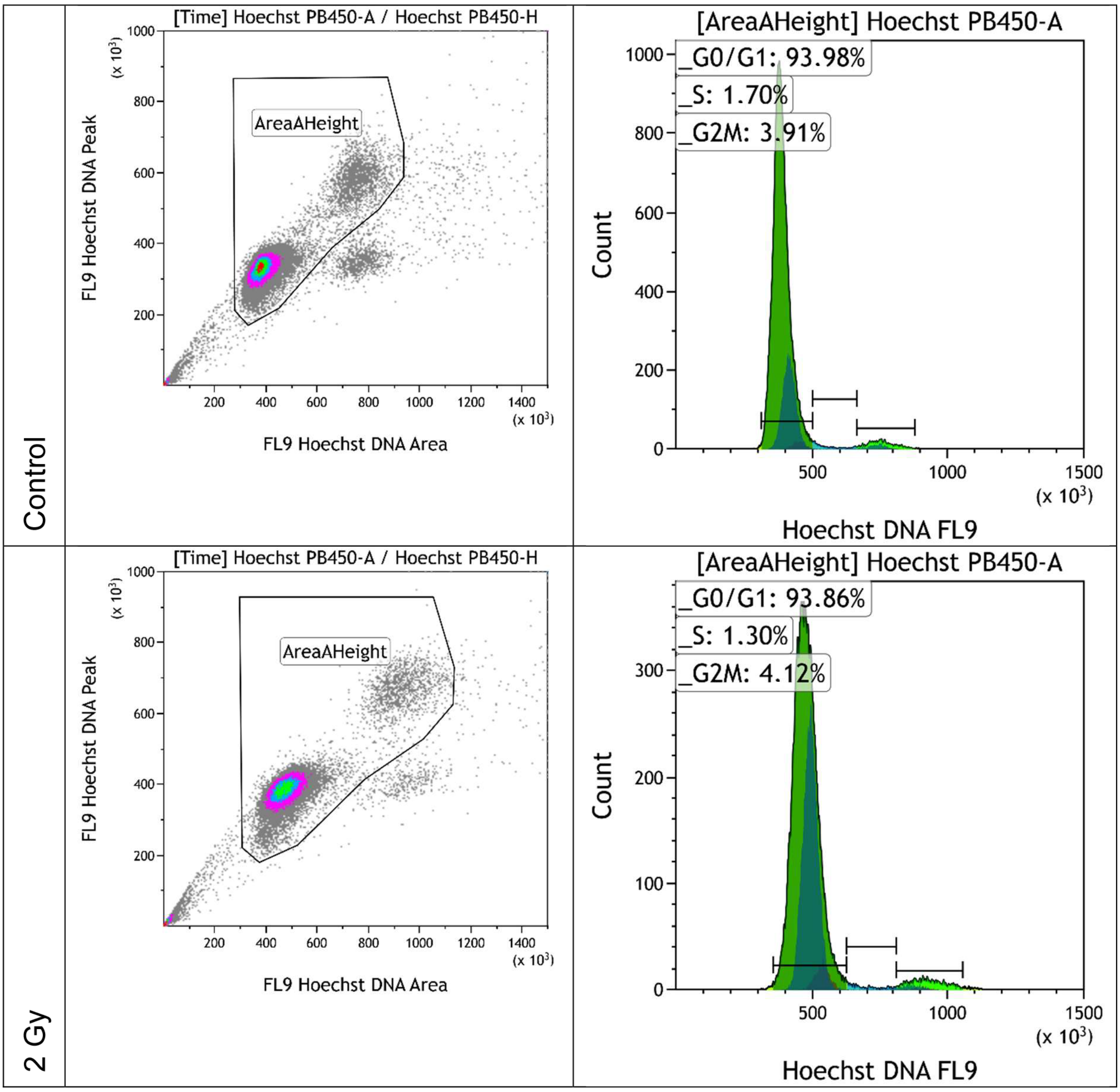

Induction of G2/M phase arrest in SBLF8

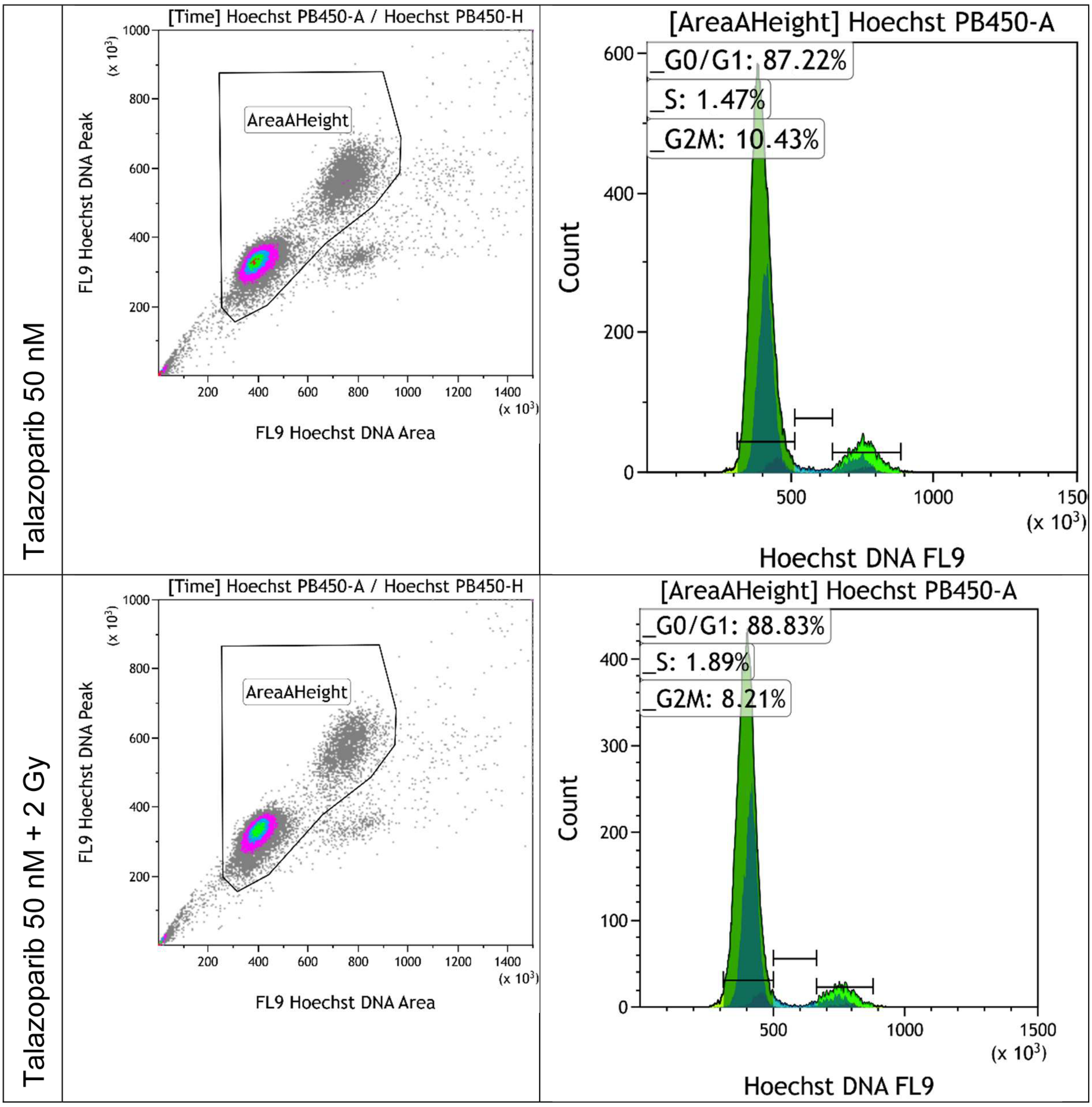

Induction of G2/M phase arrest in SBLF8

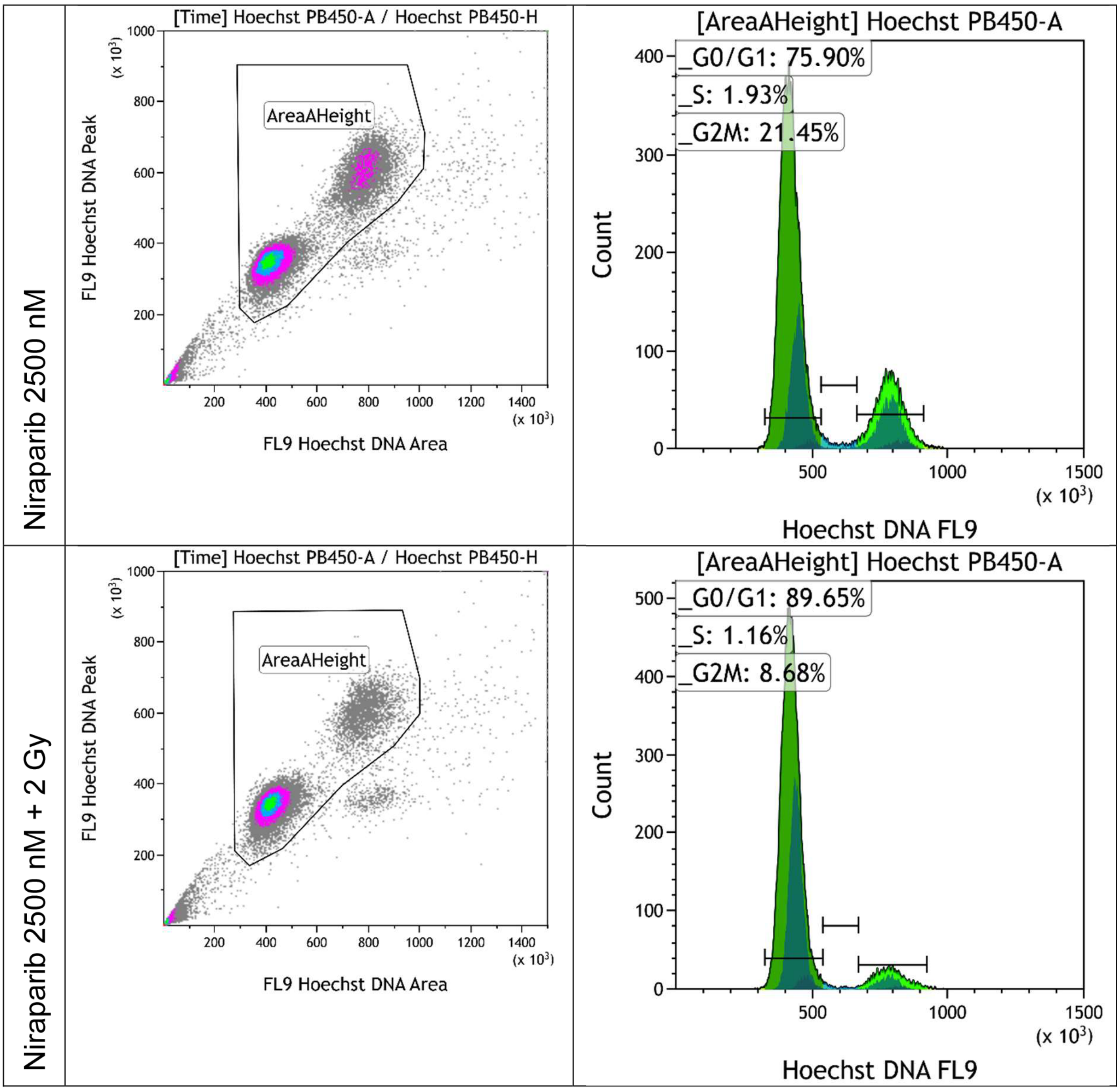

Induction of G2/M phase arrest in SBLF8

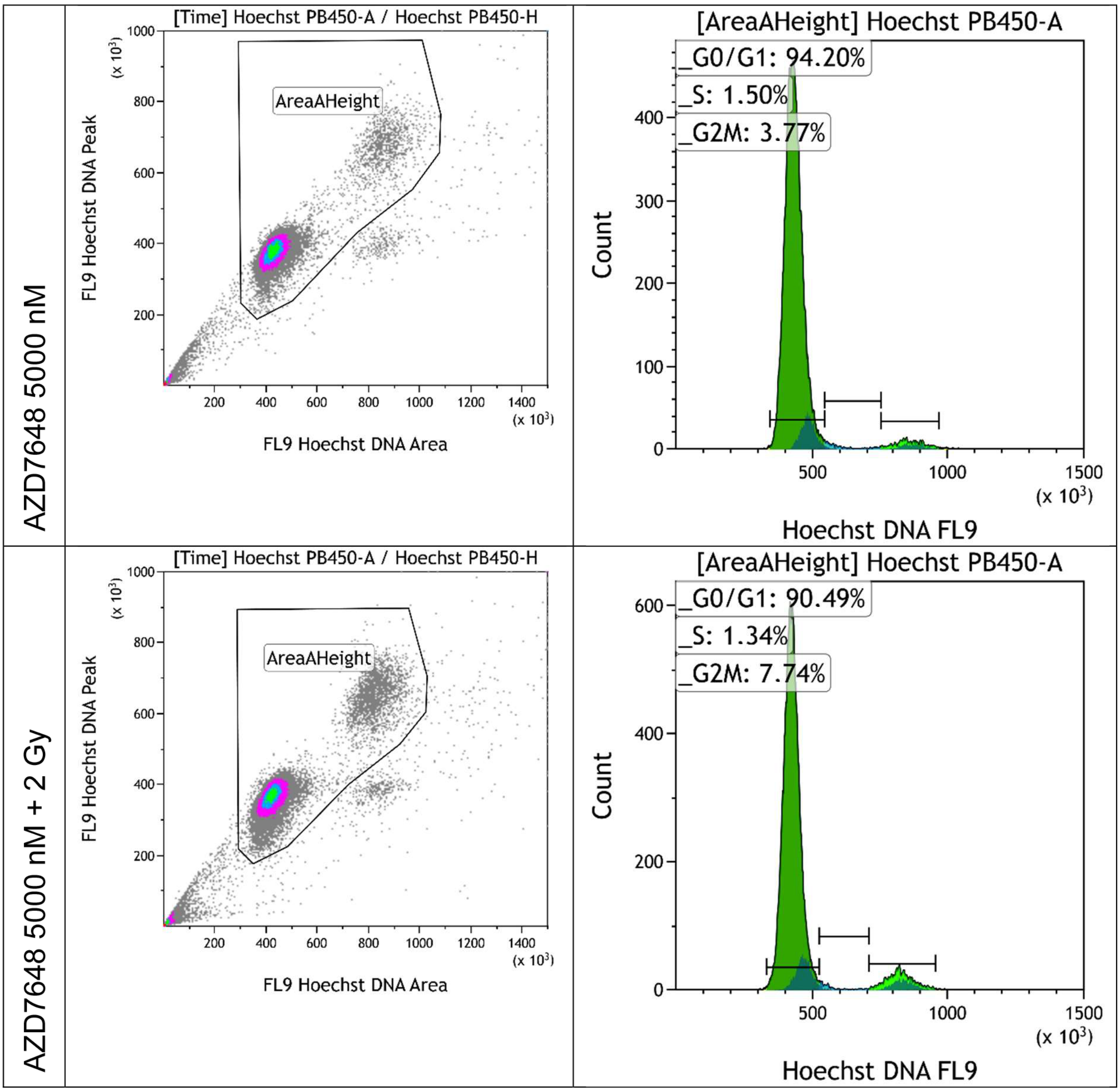

Induction of G2/M phase arrest in Cal33

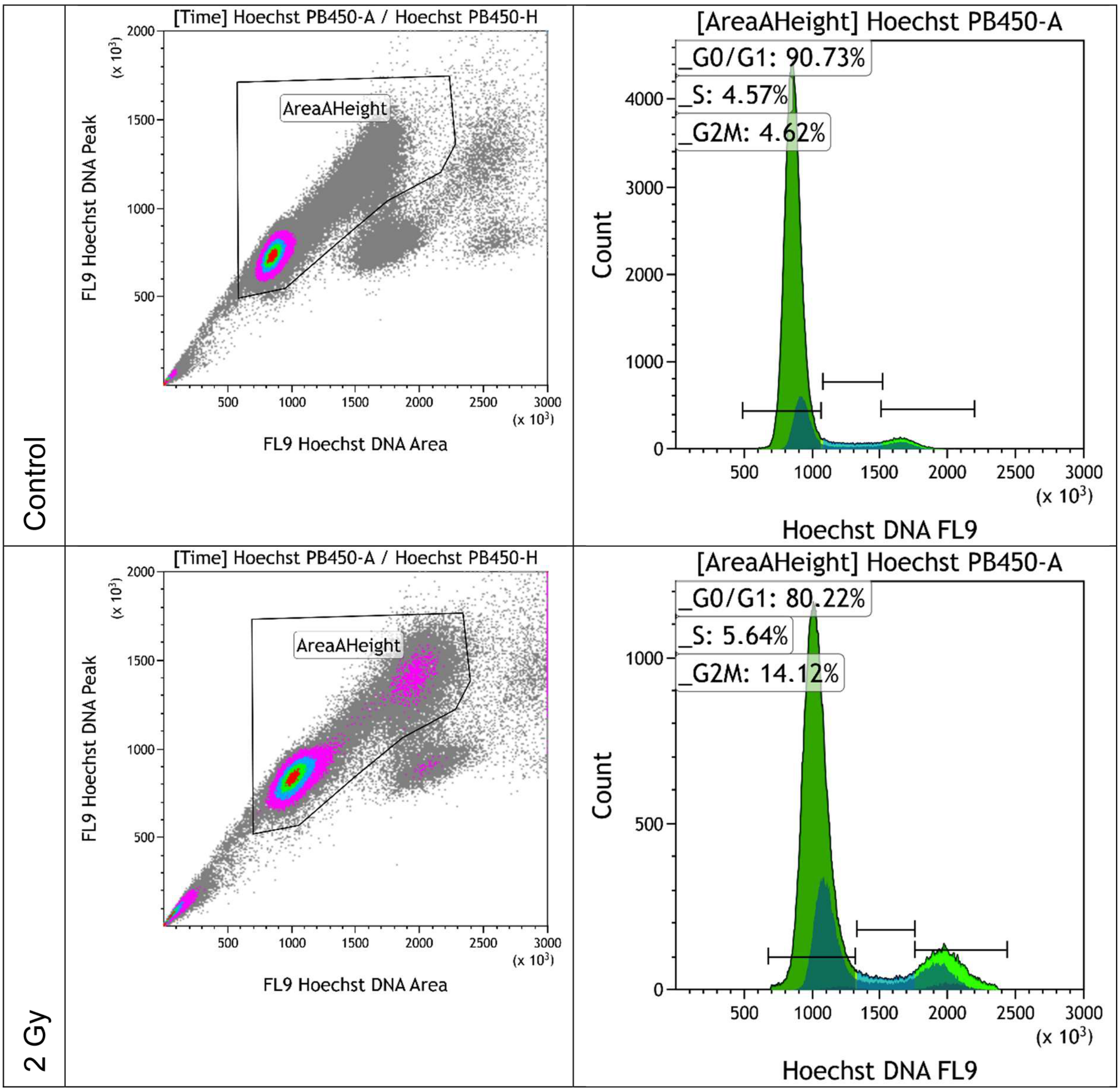

Induction of G2/M phase arrest in Cal33

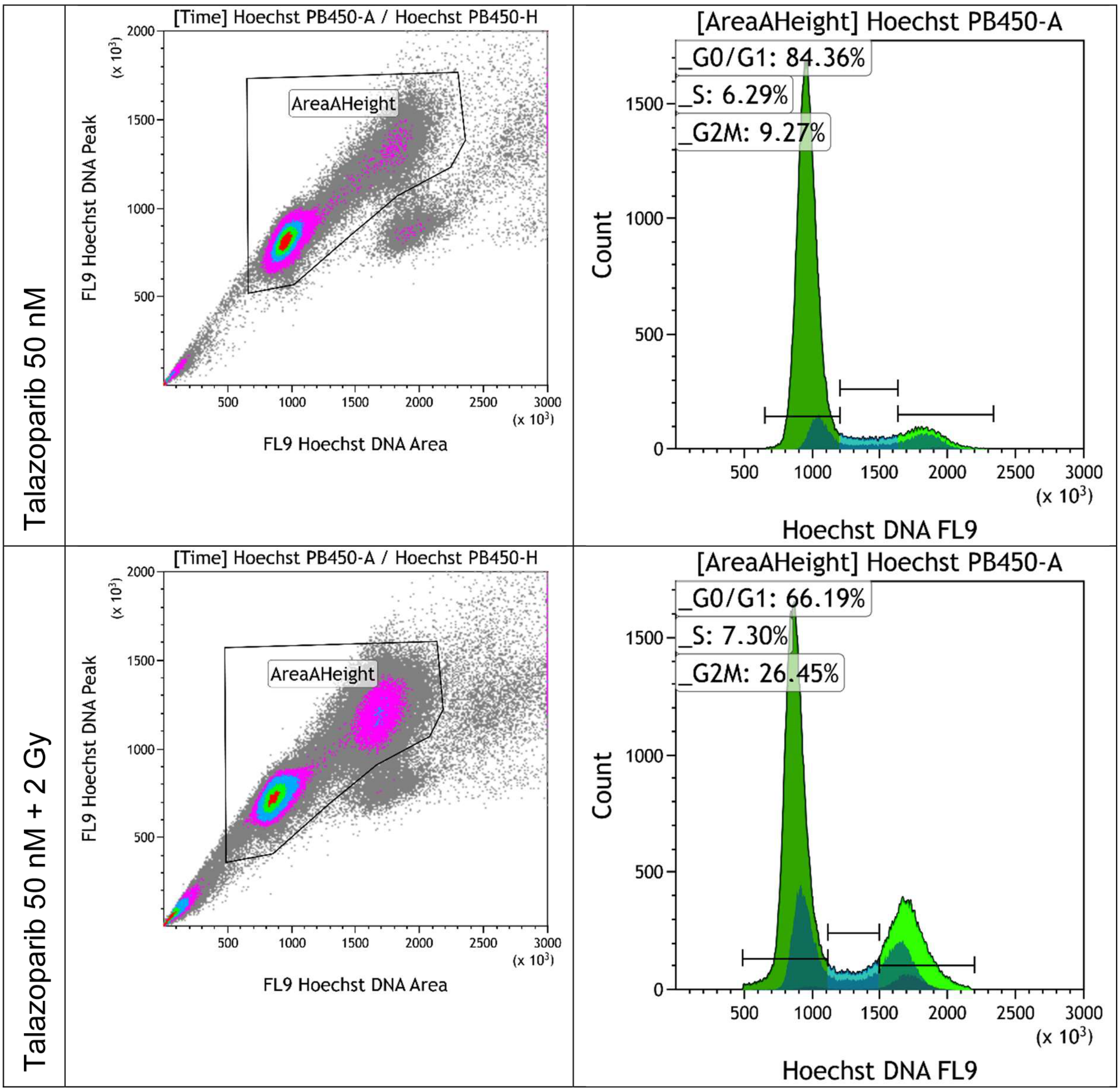

Induction of G2/M phase arrest in Cal33

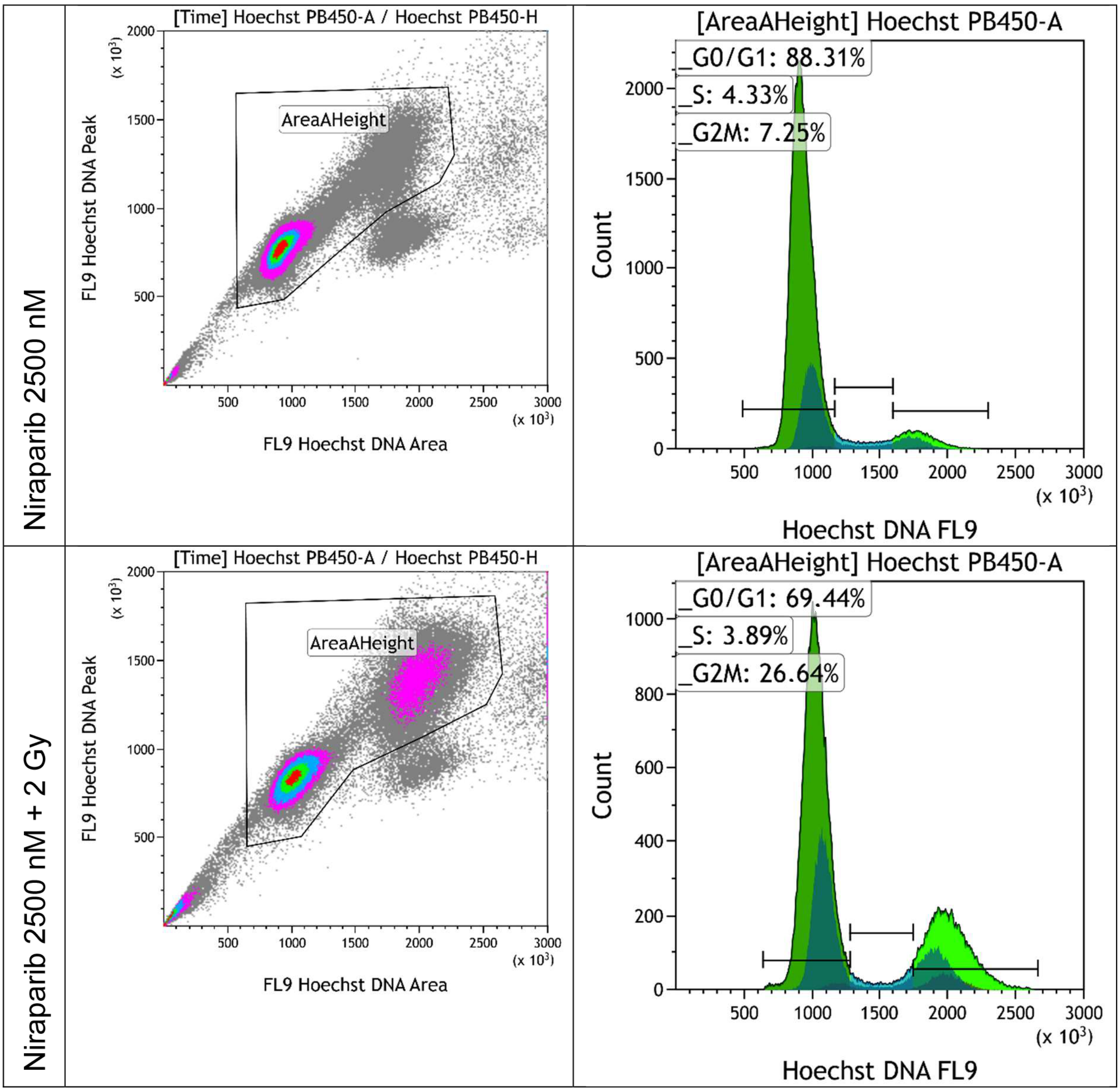

Induction of G2/M phase arrest in Cal33

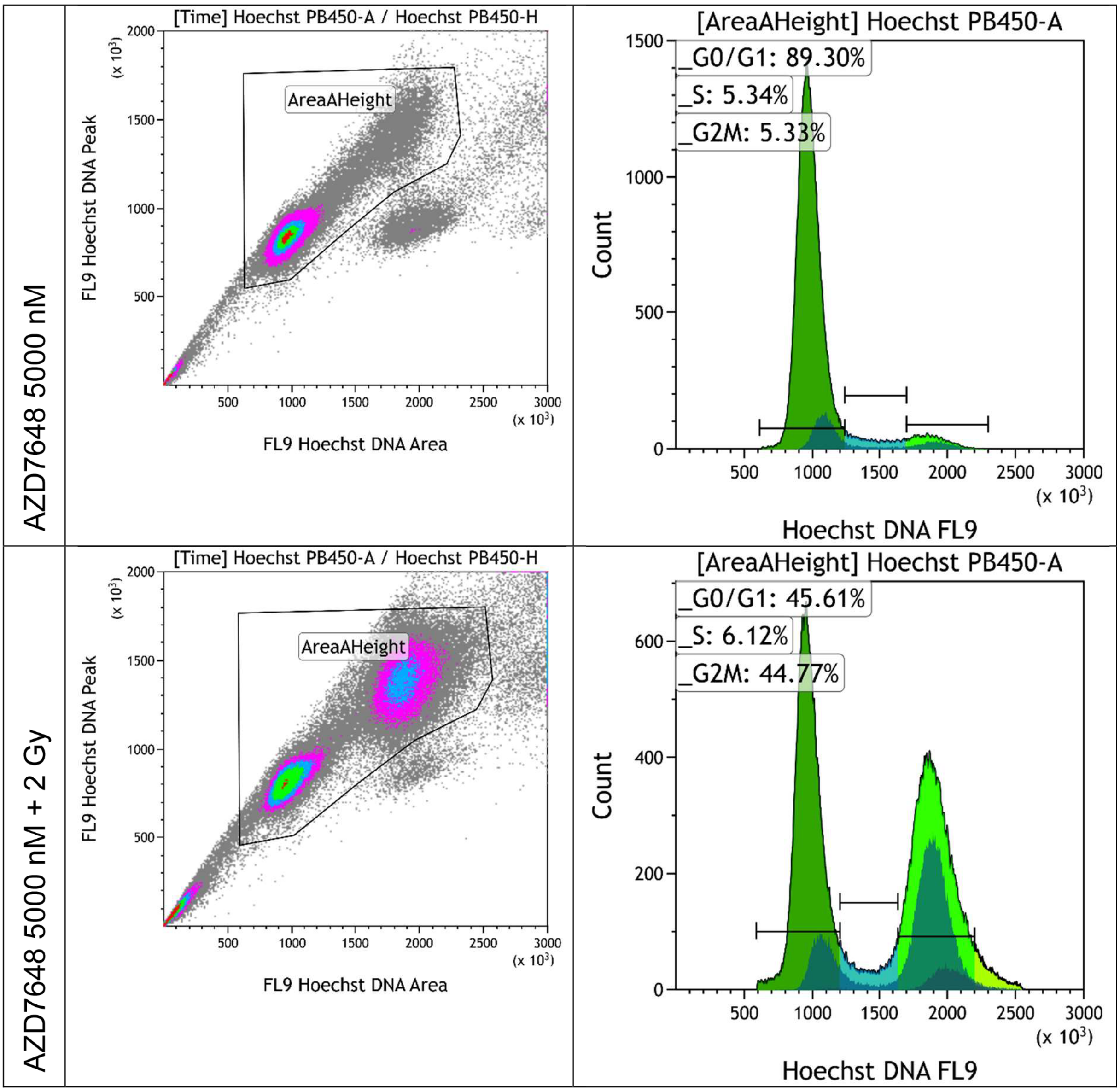

Supplement: Supplementary file 1 [file ijms-25-05629-s001.zip › Supplementary Figure S1.pdf]
