# Supplementary material for: Effective Radiosensitization of HNSCC Cell Lines by DNA-PKcs Inhibitor AZD7648 and PARP Inhibitors Talazoparib and Niraparib
Source: Int J Mol Sci. 2024 May 22;25(11):5629. doi: 10.3390/ijms25115629 (PMC11172136; doi:10.3390/ijms25115629)
Supplement: Supplementary file 1 [file ijms-25-05629-s001.zip › Supplementary Figure S2.pdf]

Supplementary Figure S2: Representative Images of Petri dishes in the Colony Formation Assay

Effective Radiosensitization of HNSCC cell lines by DNA-PKcs Inhibitor AZD7648 and PARP Inhibitors Talazoparib and Niraparib

Displayed are representative images of Petri dishes in the colony formation assay for all cell lines and treatments.

SBLF8	0 Gy	2 Gy
No inhibitor treatment	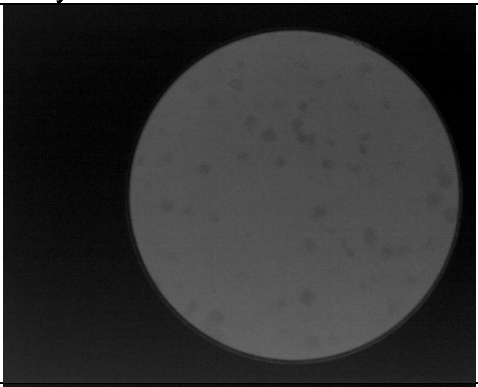	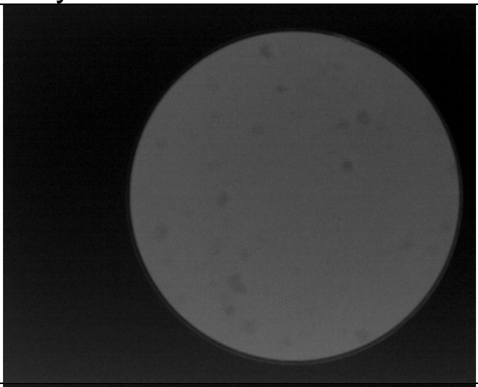
Talazoparib 50 nM	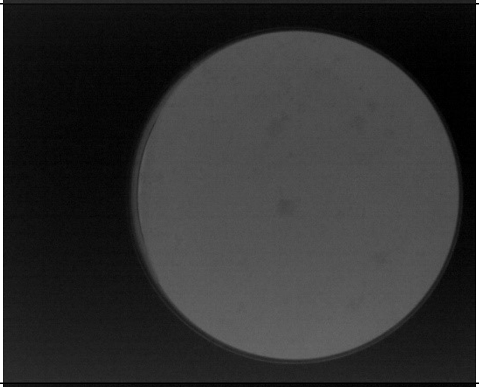	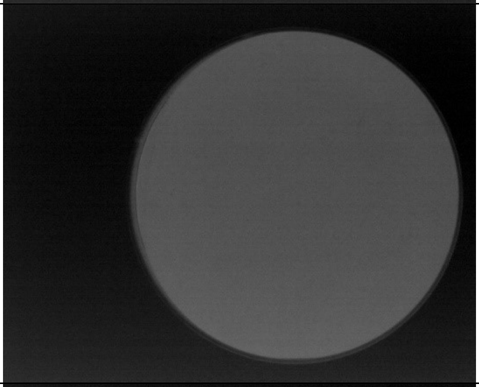
Niraparib 2500 nM	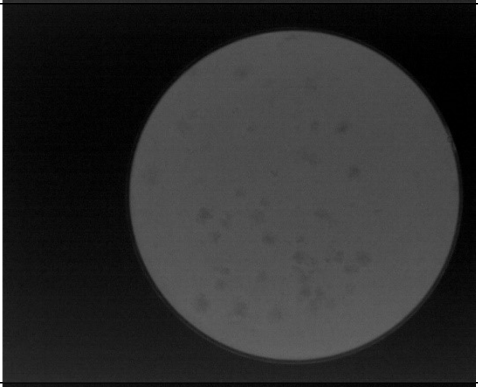	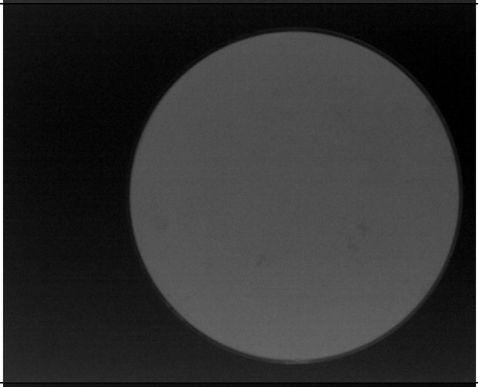
AZD7648 5000 nM	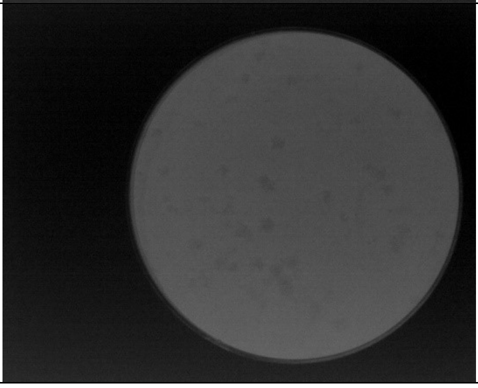	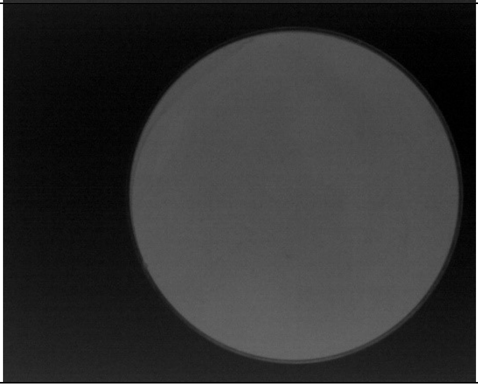

SBLF9	0 Gy	2 Gy
No inhibitor treatment	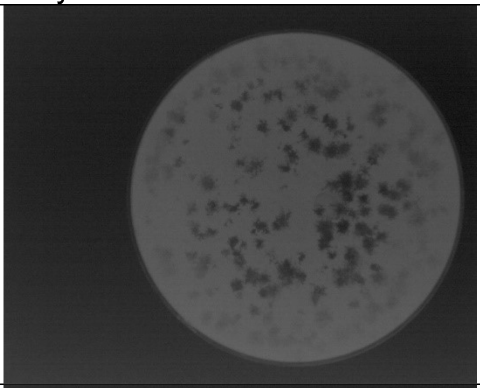	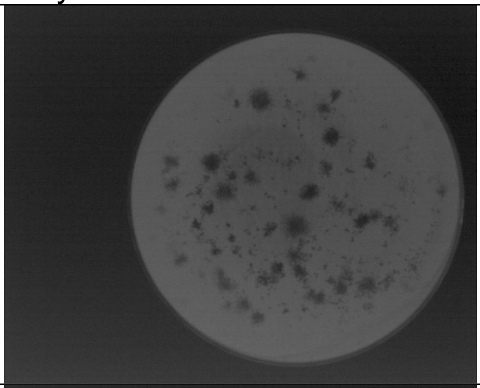
Talazoparib 50 nM	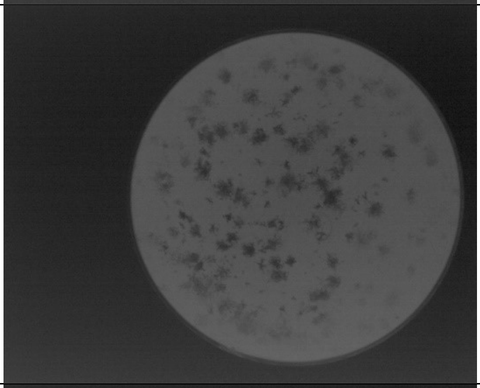	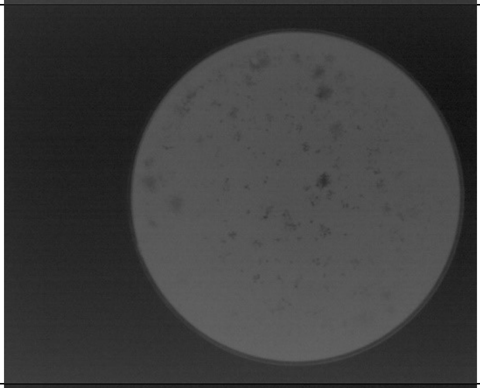
Niraparib 2500 nM	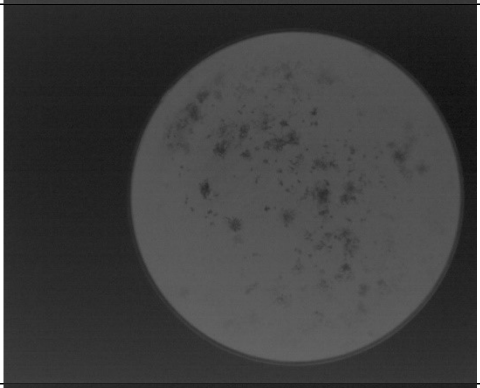	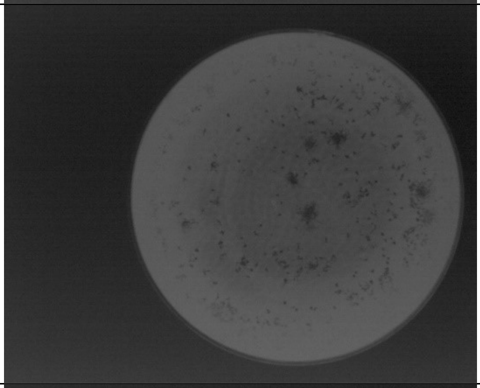
AZD7648 5000 nM	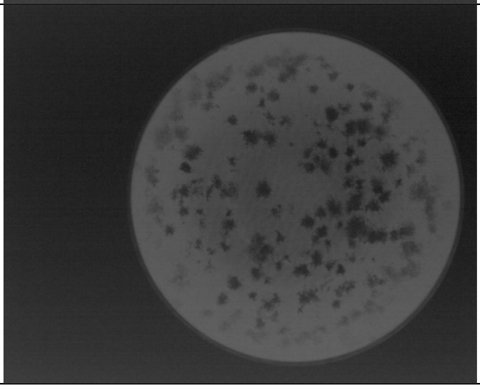	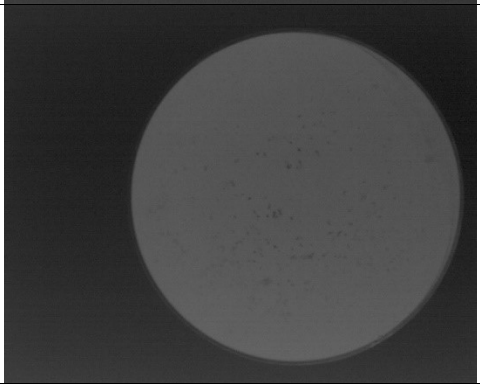

Cal33	0 Gy	2 Gy
No inhibitor treatment	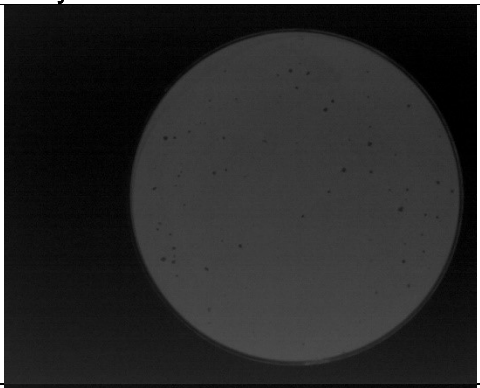	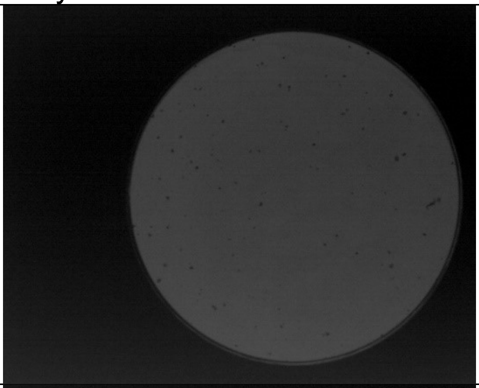
Talazoparib 50 nM	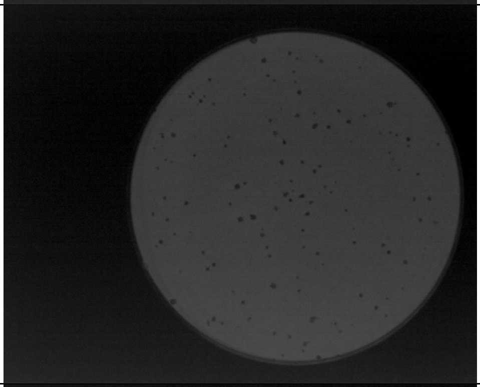	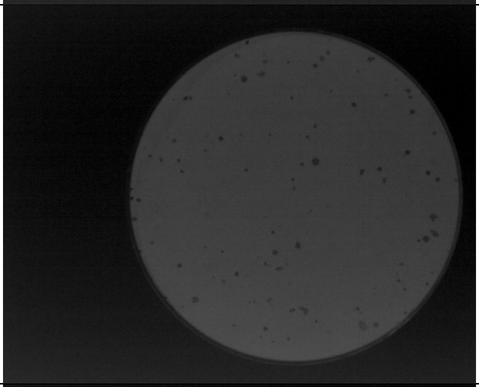
Niraparib 2500 nM	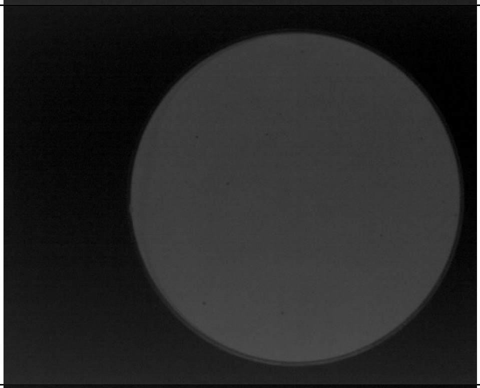	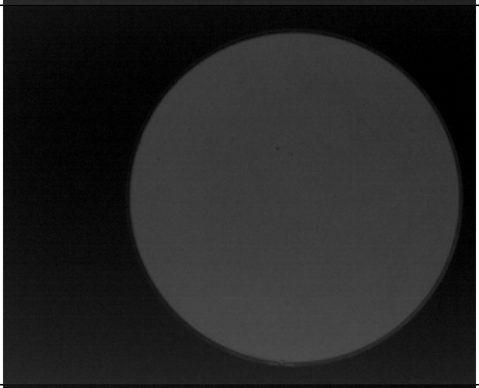
AZD7648 5000 nM	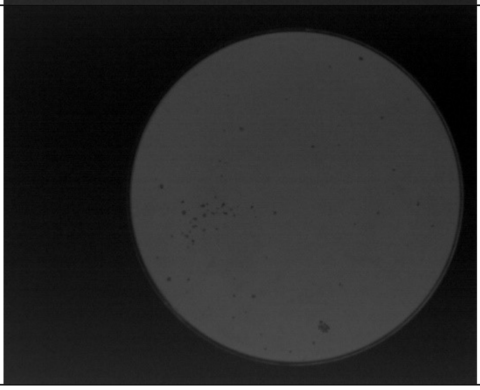	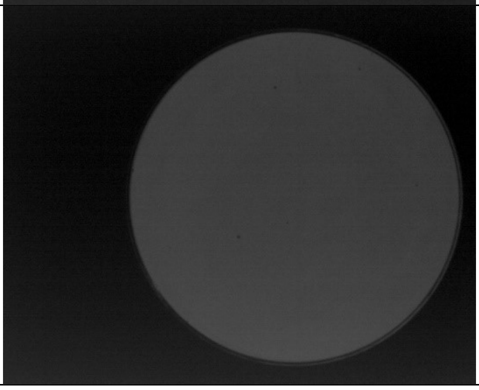

CLS-354	0 Gy	2 Gy
No inhibitor treatment	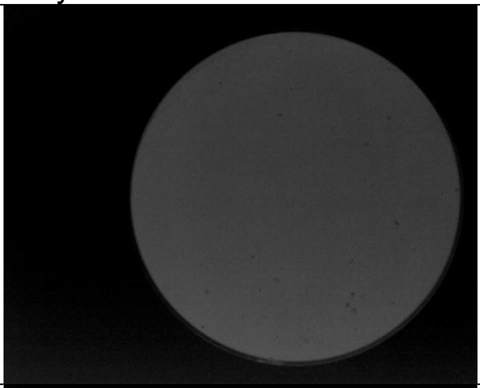	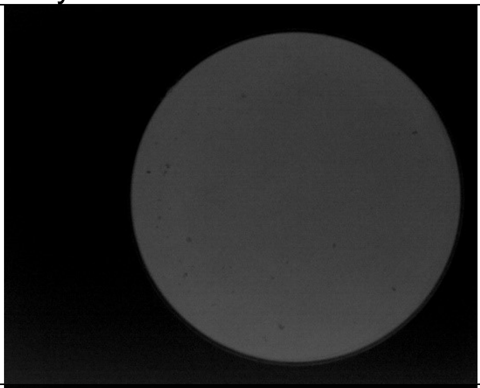
Talazoparib 50 nM	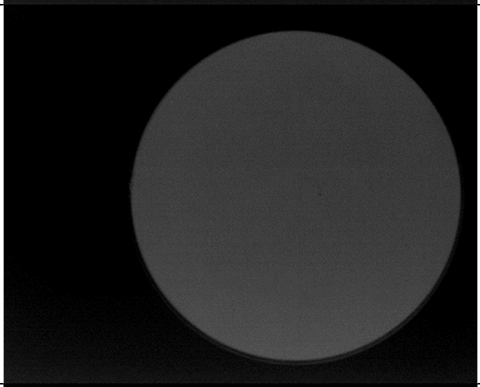	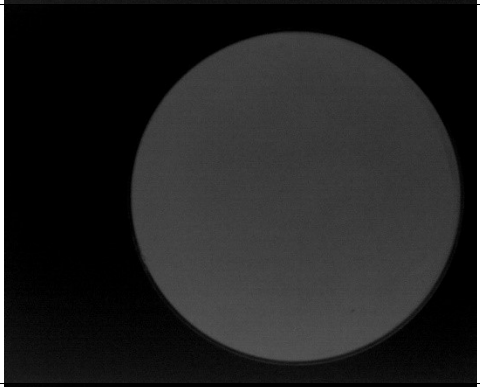
Niraparib 2500 nM	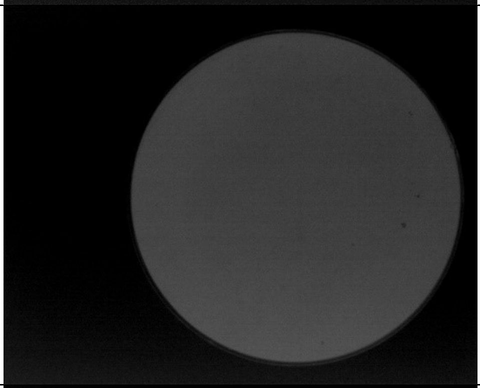	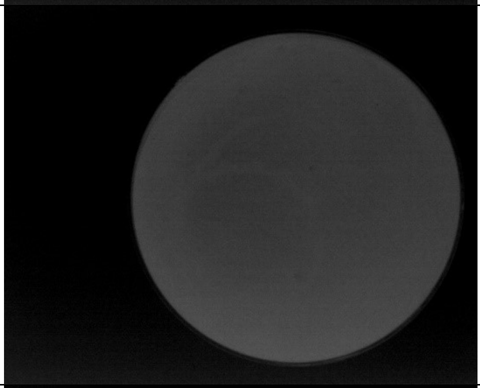
AZD7648 5000 nM	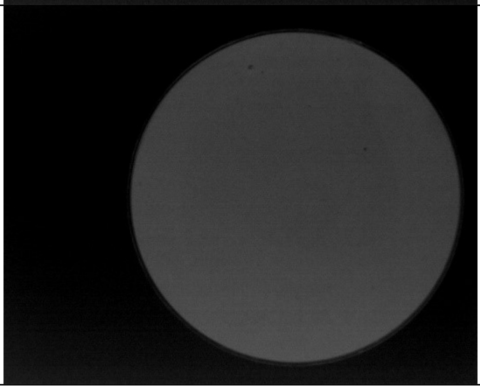	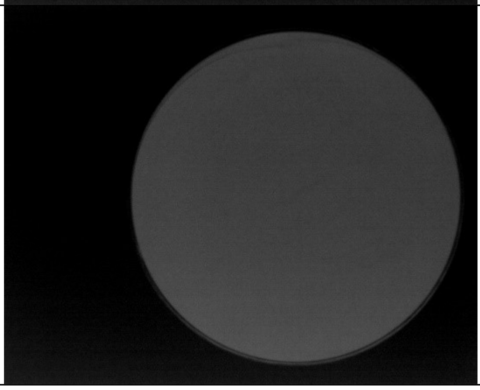

Detroit 562	0 Gy	2 Gy
No inhibitor treatment	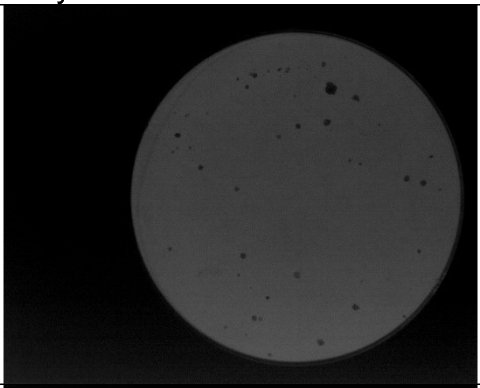	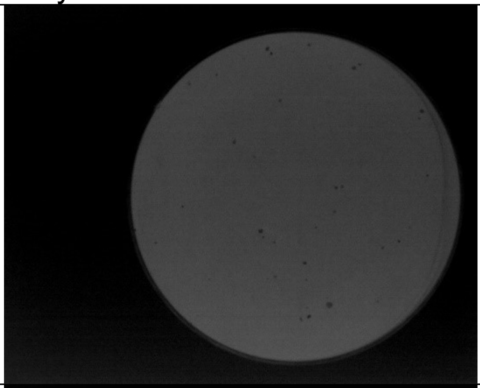
Talazoparib 50 nM	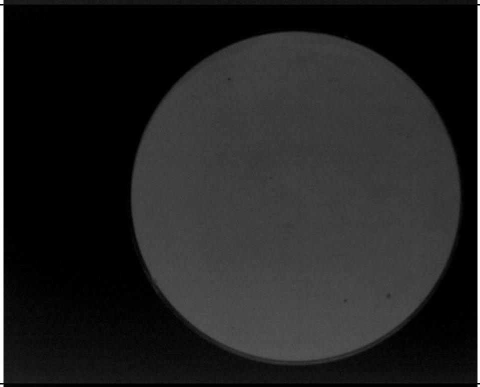	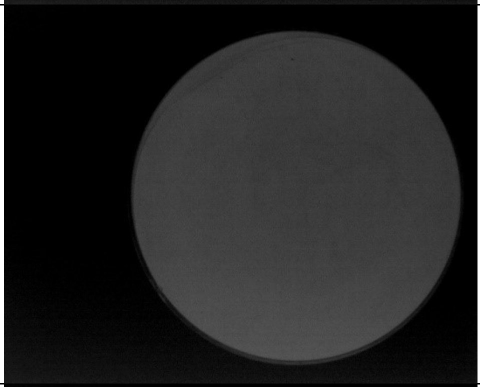
Niraparib 2500 nM	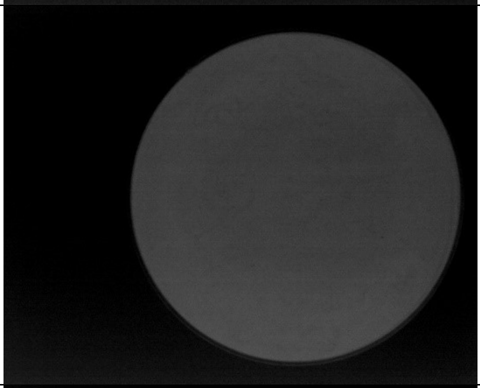	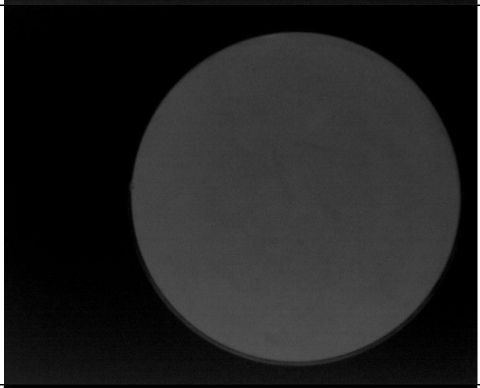
AZD7648 5000 nM	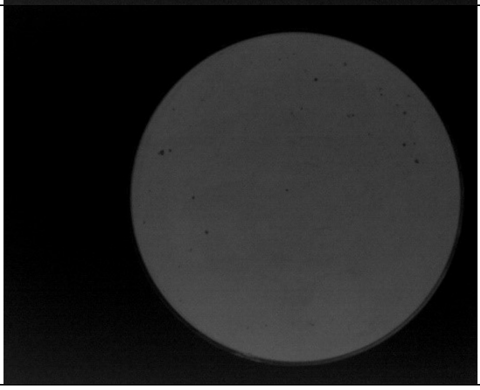	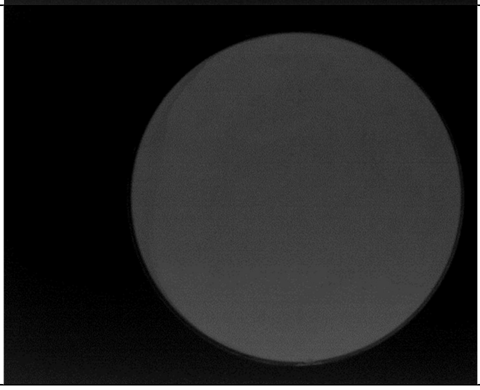

HSC4	0 Gy	2 Gy
No inhibitor treatment	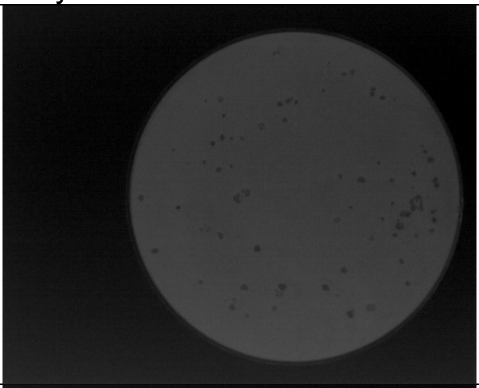	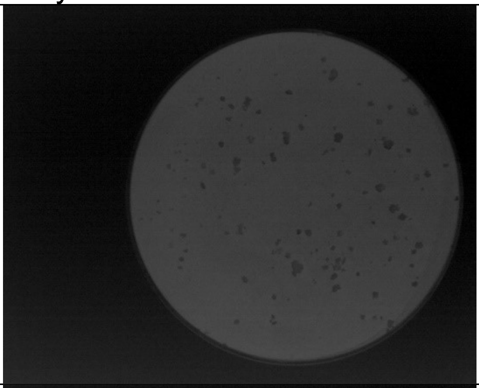
Talazoparib 50 nM	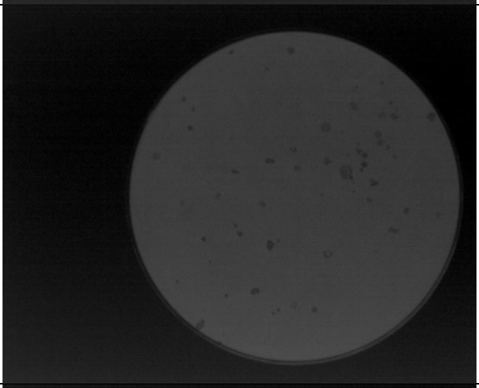	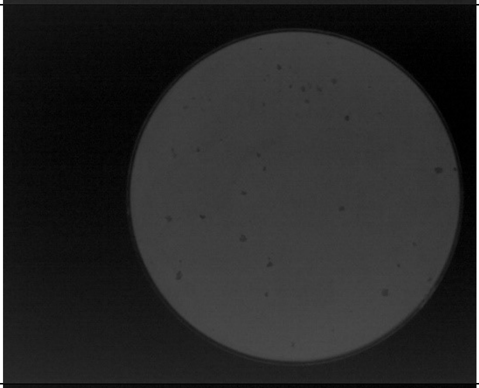
Niraparib 2500 nM	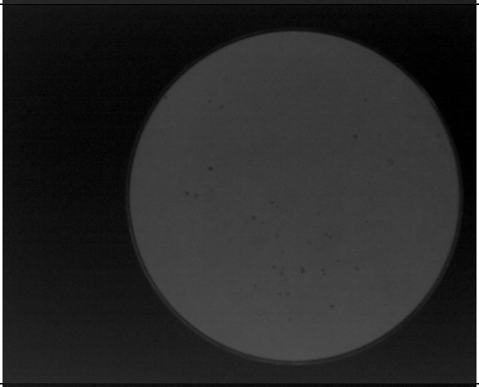	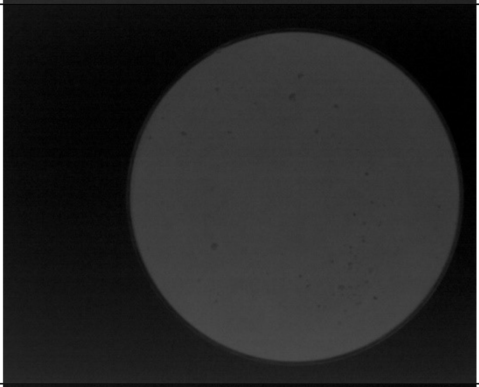
AZD7648 5000 nM	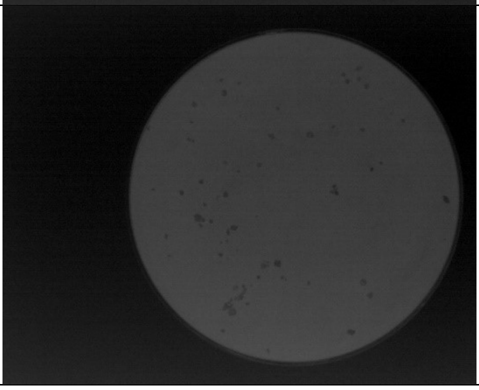	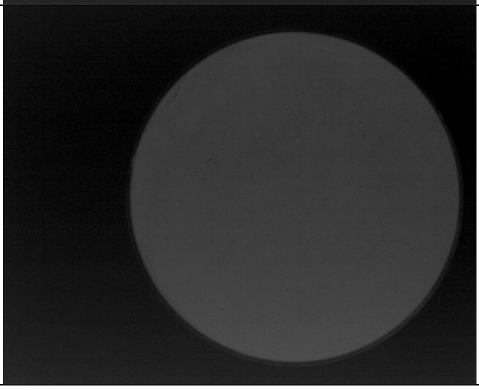

RPMI2650	0 Gy	2 Gy
No inhibitor treatment	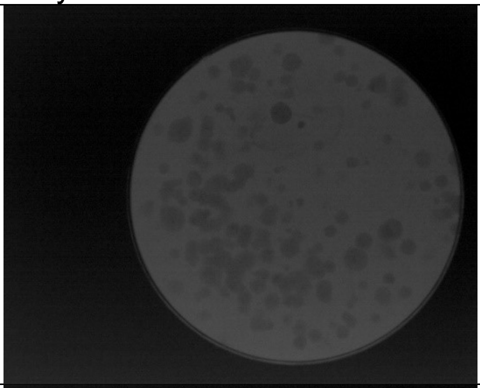	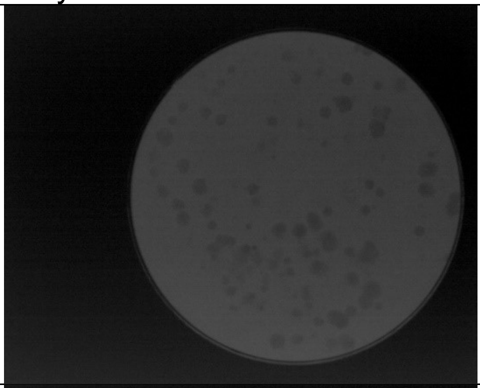
Talazoparib 50 nM	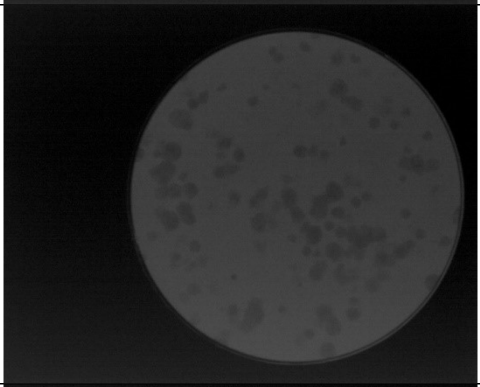	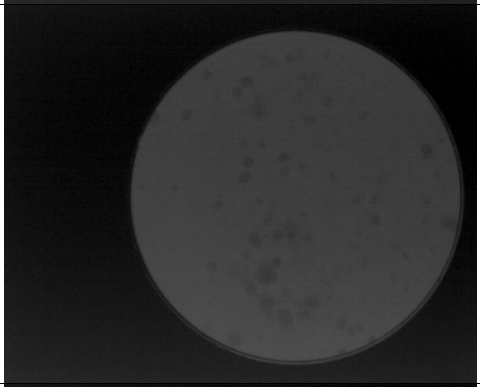
Niraparib 2500 nM	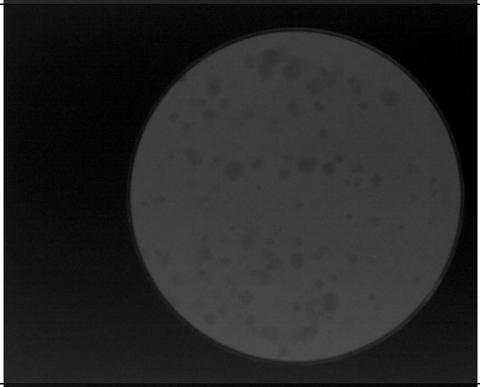	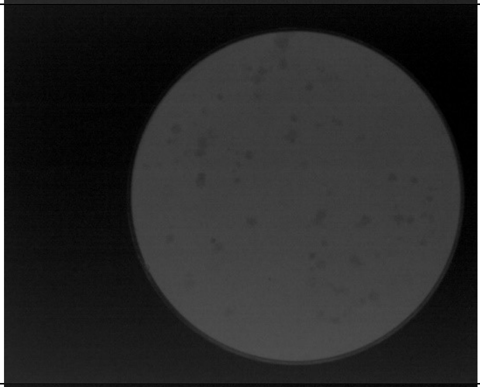
AZD7648 5000 nM	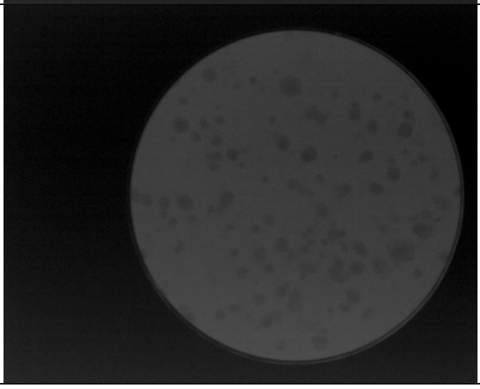	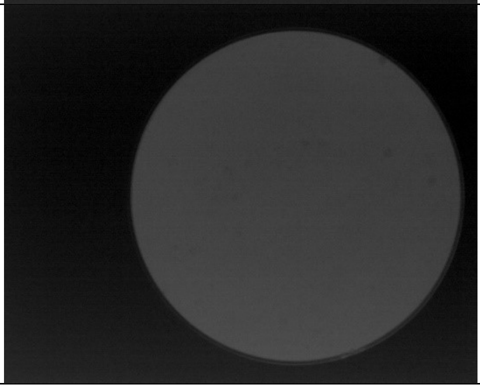

UD-SCC-2	0 Gy	2 Gy
No inhibitor treatment	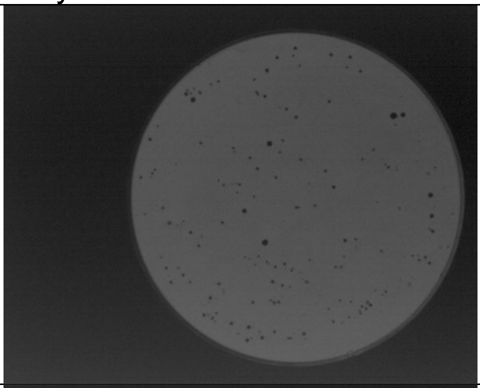	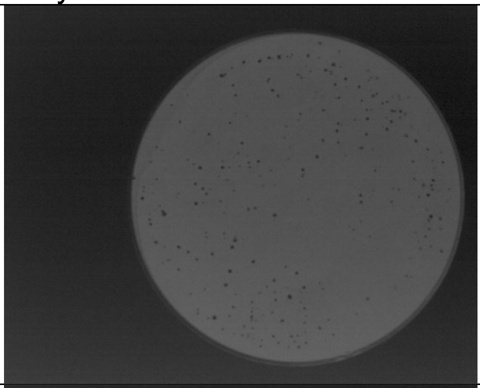
Talazoparib 50 nM	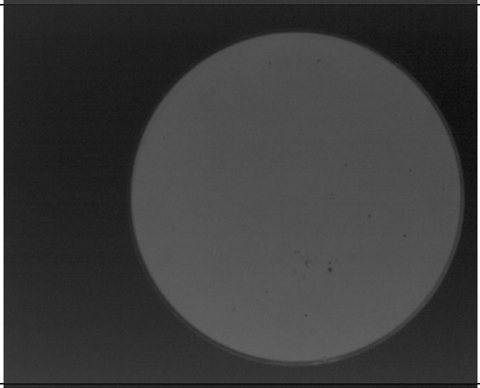	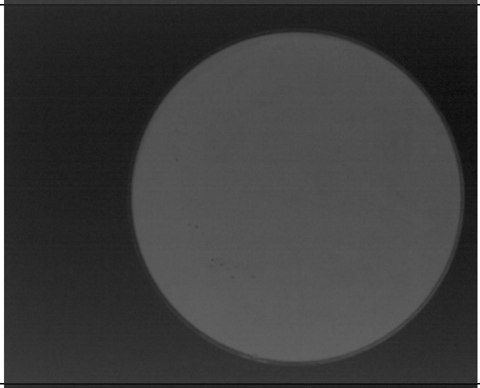
Niraparib 2500 nM	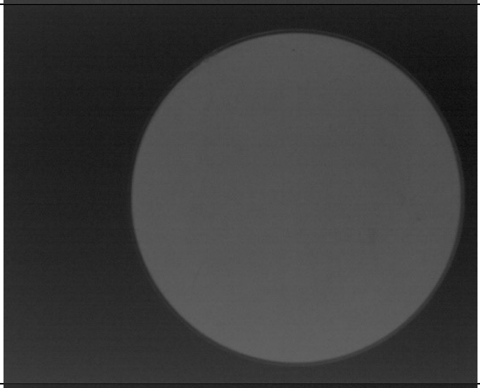	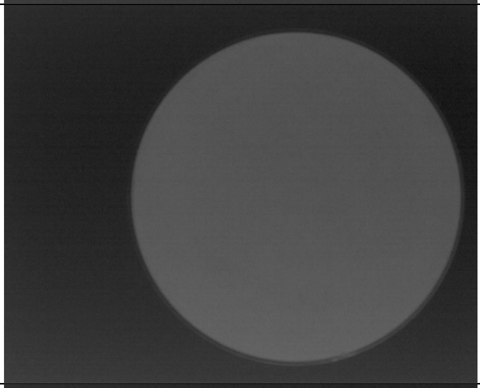
AZD7648 5000 nM	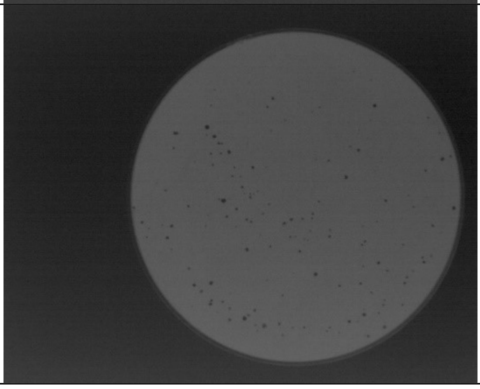	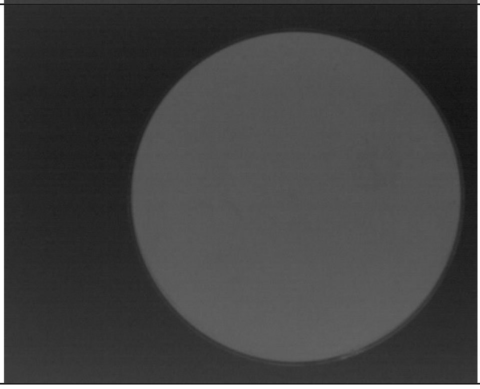

UM-SCC-47	0 Gy	2 Gy
No inhibitor treatment	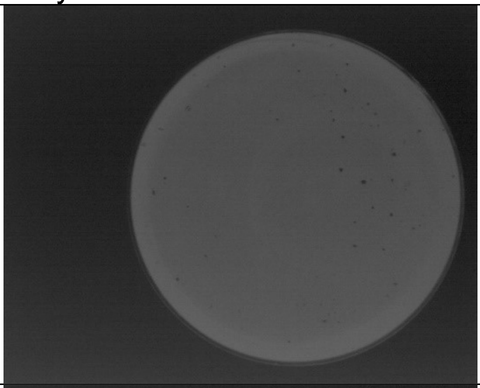	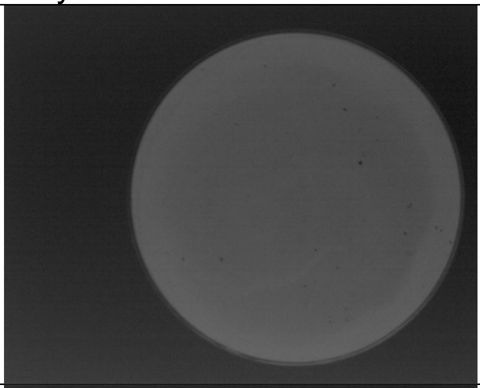
Talazoparib 50 nM	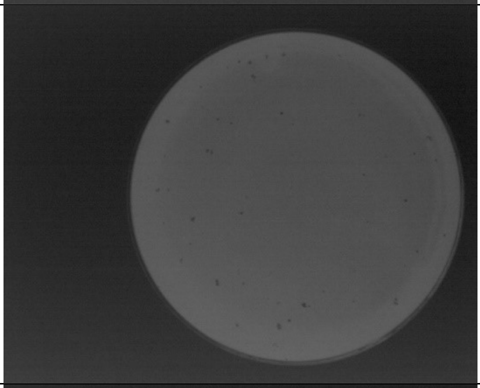	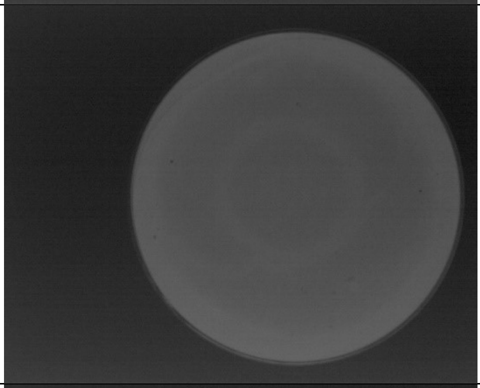
Niraparib 2500 nM	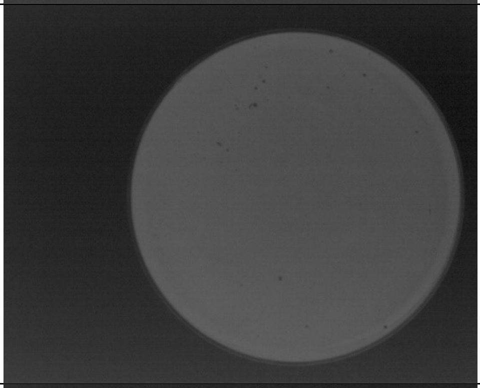	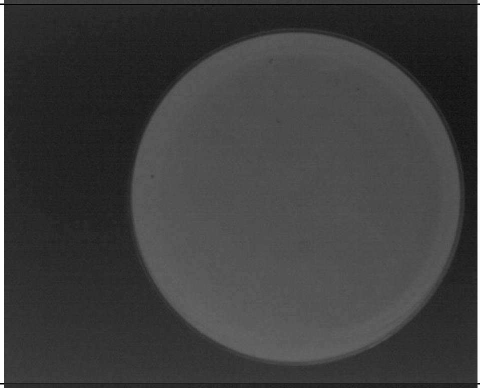
AZD7648 5000 nM	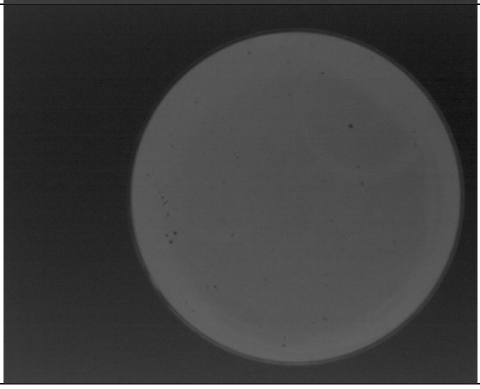	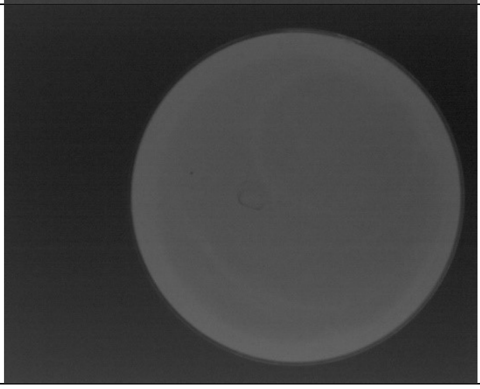
