# Supplementary material for: Effective Radiosensitization of HNSCC Cell Lines by DNA-PKcs Inhibitor AZD7648 and PARP Inhibitors Talazoparib and Niraparib
Source: Int J Mol Sci. 2024 May 22;25(11):5629. doi: 10.3390/ijms25115629 (PMC11172136; doi:10.3390/ijms25115629)
Supplement: Supplementary file 1 [file ijms-25-05629-s001.zip › Supplementary Tables S1 - S3.pdf]

Supplementary Tables S1 - S3

Effective Radiosensitization of HNSCC cell lines by DNA-PKcs Inhibitor AZD7648 and PARP Inhibitors Talazoparib and Niraparib

Supra-additivity was assessed using the unpaired, two-tailed Mann-Whitney U-test where a p-value of 0.05 or smaller determined statistically significant difference between the two groups representing supra-additivity indicated in green.

Additionally, a score was calculated from each single measurement of the two groups as described below to get a better overview of the size of the supra-additive effect. The score is presented with its mean and 95 % confidence interval. The higher the score, the higher the supra-additive effect as visually supported with color intensity of red for supra-additivity and blue for effects that are below the added effects of KI alone and IR alone.

For induction of apoptosis and necrosis and of G2/M phase arrest using values in percent, the comparison was between

Additive effect: [IR] - [Co] + [KI] - [Co]
vs.
Combination effect: [KI+IR] - [Co].

The score was created by subtracting the combination effect from the additive effect.

Supplementary Table S1: Apoptosis- and necrosis-induction: p-values and scores for supra-additivity.

	Talazoparib 50 nM and 2 Gy IR			Niraparib 2500 nM and 2 Gy IR			AZD7648 5000 nM and 2 Gy IR		
	p-value	Score		p-value	Score		p-value	Score	
		Mean	CI95%		Mean	CI95%		Mean	CI95%
SBLF8	0.056	-1.6	[-2.52; -0.69]	0.421	-1.32	[-2.29; -0.36]	0.841	-1.42	[-5.10; 2.25]
SBLF9	0.686	-1.29	[-4.04; 1.47]	0.686	-1.18	[-3.64; 1.27]	0.2	-4.3	[-8.49; -0.10]
Cal33	0.057	5.3	[1.61; 8.99]	0.114	3.06	[0.94; 5.18]	**0.029**	7.35	[3.13; 11.57]
CLS-354	**0.029**	13.84	[9.74; 17.94]	0.2	3.81	[-0.64; 8.26]	**0.029**	21.66	[16.68; 26.65]
Detroit 562	0.686	1.76	[-1.69; 5.21]	0.486	1.25	[-0.31; 2.82]	**0.029**	9.34	[3.23; 15.45]
HSC4	0.343	3.34	[1.18; 5.49]	**0.029**	9.45	[7.17; 11.73]	0.114	16.69	[8.48; 24.89]
RPMI2650	>0.9999	-0.65	[-3.50; 2.19]	0.886	0.59	[-1.05; 2.23]	0.114	3.5	[-0.26; 7.26]
UD-SCC-2	0.4	4.96	[-1.59; 11.51]	>0.9999	-0.04	[-10.37 ;10.28]	0.2	11.23	[7.61; 14.84]
UM-SCC-47	0.057	4.39	[2.65; 6.13]	0.114	2.83	[0.50; 5.15]	**0.029**	14.43	[11.19; 17.67]

CI95% = 95 % confidence interval

Supplementary Table S2: G2/M phase arrest induction: p-values and scores for supra-additivity.

	Talazoparib 50 nM and 2 Gy IR			Niraparib 2500 nM and 2 Gy IR			AZD7648 5000 nM and 2 Gy IR		
	p-value	Score		p-value	Score		p-value	Score	
		Mean	CI95%		Mean	CI95%		Mean	CI95%
SBLF8	0.222	-2.13	[-2.69; -1.57]	0.008*	-14.33	[-19.86 ; -8.81]	0.056	2.09	[0.56; 3.62]
SBLF9	0.486	1.87	[1.30; 2.43]	0.686	-0.82	[-3.29; 1.65]	0.486	2.37	[-0.16; 4.90]
Cal33	**0.029**	7.65	[6.66; 8.65]	0.114	10.2	[5.11; 15.29]	**0.029**	34.17	[21.70; 46.65]
CLS-354	0.2	-3.81	[-6.69; -0.92]	0.343	-3.14	[-5.95; -0.32]	**0.029**	43.36	[40.52; 46.20]
Detroit 562	**0.029**	14.32	[12.52; 16.12]	>0.9999	0.08	[-3.53; 3.70]	**0.029**	25.96	[23.95; 27.96]
HSC4	0.343	3.65	[1.32; 5.98]	**0.029**	8.61	[4.19; 13.02]	**0.029**	36.47	[25.28; 47.65]
RPMI2650	**0.029**	16.21	[9.12; 23.29]	**0.029**	6.59	[0.21; 12.96]	**0.029**	51.24	[46.30; 56.18]
UD-SCC-2	**0.029**	26.2	[24.63; 27.77]	0.057	17.23	[15.72; 18.74]	**0.029**	59.37	[56.43; 62.30]
UM-SCC-47	**0.029**	11.02	[8.64; 13.40]	0.114	5.68	[4.94; 6.42]	**0.029**	48.31	[42.68; 53.94]

CI95% = 95 % confidence interval

** p-value does not represent supra-additivity since the added effects are significantly greater than the combined effect of 2500 nM Niraparib and 2 Gy IR.*

For colony formation assay SF was used and the comparison was between

[IR]
vs.
[KI + IR] / [KI].

The score was created by dividing [IR] by [KI + IR] / [KI].

Supplementary Table S3: Cell inactivation in the colony formation assay: p-values and scores for supra-additivity.

	Talazoparib 50 nM and 2 Gy IR			Niraparib 2500 nM and 2 Gy IR			AZD7648 5000 nM and 2 Gy IR		
	p-value	Score		p-value	Score		p-value	Score	
		Mean	CI95%		Mean	CI95%		Mean	CI95%
SBLF8	**0.029**	6.27	[1.35; 11.19]	0.057	2.12	[1.34; 2.90]	**0.029**	40.53	[21.29; 59.77]
SBLF9	**0.029**	3.80	[0.36; 7.25]	0.343	5.46	[0.24; 10.68]	**0.029**	27.59	[-2.13; 57.32]
Cal33	0.2	1.33	[0.96; 1.70]	0.343	2.56	[0.19; 4.92]	**0.029**	29.84	[13.05; 46.64]
CLS-354	0.114	*4.39**	*[;]**	0.114	1.90	[0.97; 2.84]	**0.029**	*25.30**	*[;]**
Detroit 562	0.343	2.65	[0.46; 4.85]	0.314	*2.58**	*[;]**	**0.029**	122.35	[16.13; 228.57]
HSC4	**0.029**	1.59	[1.10; 2.08]	0.057	1.47	[1.13; 1.81]	**0.029**	53.81	[4.08; 103.53]
RPMI2650	**0.029**	1.46	[1.17; 1.76]	0.057	1.44	[1.04; 1.83]	**0.029**	77.87	[-49.85 ;205.60]
UD-SCC-2	**0.029**	5.15	[2.47; 7.83]	**0.029**	2.04	[1.34; 2.74]	**0.029**	230.96	[57.22; 404.71]
UM-SCC-47	**0.029**	2.10	[1.34; 2.87]	**0.029**	2.07	[1.39; 2.75]	**0.029**	20.23	[12.31; 28.15]

CI95% = 95 % confidence interval

** Score calculation would have required division by 0. The score displayed is not the mean of four individual score calculations but one score calculation from the mean values of the groups and the calculation of CI95% is not applicable.*
